# From Beam Damage to Massive Reaction Amplification under the Electron Microscope: An Ionization-Induced Chain Reaction in Crystals of a Dewar Benzene

**DOI:** 10.1021/acscentsci.4c01429

**Published:** 2024-12-06

**Authors:** Krzysztof A. Konieczny, Indrajit Paul, Jose A. Rodriguez, Miguel A. Garcia-Garibay

**Affiliations:** † Department of Chemistry and Biochemistry, University of California at Los Angeles, Los Angeles, California 90095, United States; ‡ Faculty of Chemistry, Wrocław University of Science and Technology, Wybrzeże Wyspiańskiego 27, 50-370 Wroclaw, Poland

## Abstract

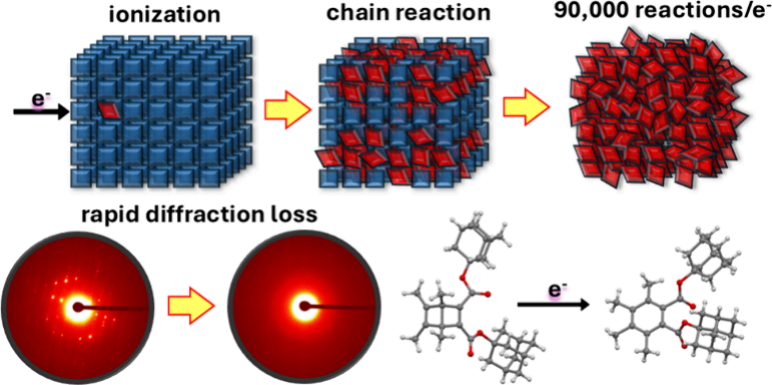

Electron microscopy in its various forms is one of the most powerful imaging and structural elucidation methods in nanotechnology where sample information is generally limited by random chemical and structural damage. Here we show how a well-selected chemical probe can be used to transform indiscriminate chemical damage into clean chemical processes that can be used to characterize some aspects of the interactions between high-energy electron beams and soft organic matter. Crystals of a Dewar benzene exposed to a 300 keV electron beam facilitate a clean valence-bond isomerization radical-cation chain reaction where the number of chemical events per incident electron is amplified by a factor of up to ca. 90,000.

## Abstract 2

A crystalline Dewar benzene undergoes up to 90,000 reactions per incident electron, rapidly losing diffraction and massively amplifying the effects of ionization in microelectron diffraction.

Electron beams used in modern electron microscopy imaging and diffraction measurements cause significant damage to soft organic and biological materials.^1^ Scattered experimental evidence suggests that damage is initiated by inelastic electron scattering processes where a fraction of the beam energy results in ionization, bond-cleavage, knock-on atomic and molecular displacements, heating, and secondary electrons.^2,3^ While degradation is unavoidable, structural determination by diffraction analysis becomes possible by mitigating electron capture^4^ with thin crystals (ca. 100–300 nm),^5^ low density flux beams (0.01–0.05 e^–^/Å^2^ s), and high energy electrons (ca. 200–300 keV), such that microelectron diffraction (micro-ED),^6^ has become a powerful tool for structural elucidation of submicron size crystals. While a detailed understanding of chemical damage would be highly desirable, product analysis is limited by total electron beam fluence of only ∼1 e^–^/Å^2^,^4^ which correspond to less than one incident electron per molecule, combined with the fact that most electrons are either transmitted unaffected or coherently diffracted. However, based on our recent experience on signal amplification in crystalline solids by selecting adiabatic reactions and excitons as chain carriers,^7,8^ we recognized the potential of using crystals built with molecules prone to radical-ion skeletal rearrangements to set up chain reactions that may lead to thousands of product molecules per ionization, potentially becoming an ideal approach to test for ionization-based damage, and exploring applications in materials science, sensing, and space-resolved nanochemical synthesis.

As a test system, we explored the radical cation valence-bond isomerization in crystals of Dewar benzene 3,4,5,6-tetramethyl-1,2-diadamantyl-dicarboxylate **1**, expected to transform upon exposure to the electron beam into benzene 3,4,5,6-tetramethyl-1,2-diadamantyl-dicarboxylate **2** (Figure 1B). The first step is an e-beam induced ionization by removing an electron from Dewar benzene (**1**) to generate radical cation **1**^•+^ (Figure 1A,C). Chain propagation relies on rearrangement of **1**^•+^ to benzene radical cation (**2**^•+^, Step 2), followed by electron transfer from another **1** (Step 3), which leads to the formation of product **2** and a new radical cation **1**^•+^ ready to enter a new cycle. Chain propagation occurs by multiple iterations of Steps 2 and 3, and chain termination by reaction with unspecified electron donor (ED) impurities (Step 4), or by radical–radical bond formation to generate dications (not shown).

**Figure 1:**
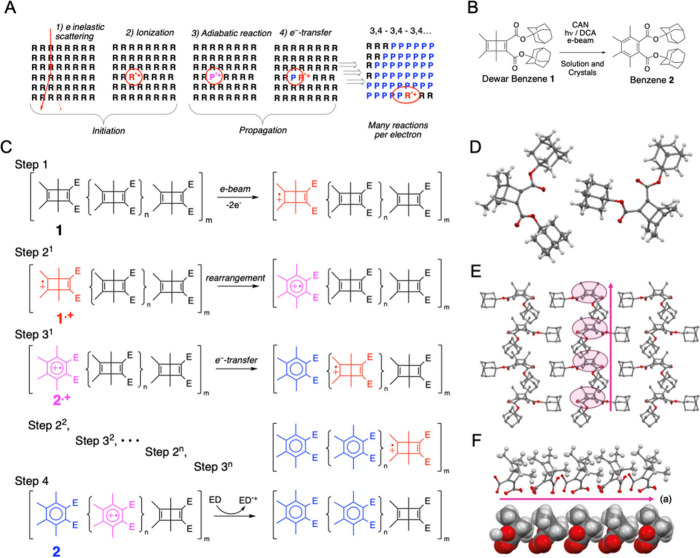
**e-Beam initiated chain reaction**. (A) Steps involved in product amplification by a radical-ion chain reaction. (B) Valence-bond isomerization of Dewar benzene by a radical cation chain reaction initiated by single electron oxidation using ceric ammonium nitrates (CAN), ultraviolet light with 9,10-dicyanoanthracene as a photooxidant, and the electron-beam of an electron microscope. (C) Steps involved in the reaction mechanism of the radical cation chain reaction. (D) Asymmetric unit in crystals of Dewar benzene **1**. (E) Packing arrangement of **1** illustrating the segregation of Dewar benzene and adamantane groups. (F) Dewar benzenes with ball and stick and space-filling models with the ester groups removed to illustrates the infinite chains with close van der Waals contacts along the crystallographic *a* direction.

Radical cation rearrangements of hexamethyl Dewar benzene have been documented in cryogenic matrices,^9,10^ and chain reactions in polar solvents.^11^ The release of ring strain and gained aromaticity render the valence-bond isomerization from **1** to **2** thermodynamically favorable by ca. 60 kcal/mol.^12,13^ In terms of electron transfer parameters, an early cyclic voltammetry study reports that oxidation of Dewar benzene and benzene in acetonitrile are essentially identical, with values of 1.58 V for **1** and 1.62 V for **2**, vs SCE (10), suggesting that electron transfer could be reversible and inefficient. However, experimental^14^ and theoretical (13) work support strongly unidirectional hole transfer from **2**^•+^ to **1** based on the large difference in reorganization energy (λ) required in going from **1**^•+^ to **1** as compared to **2**^•+^ to **2** (Δλ ∼ 0.63 eV). Matrix isolation experiments and calculations^9,10^ indicate that reaction starts from a π-radical cation with the electron hole localized in one of the π-bonds, which transforms into a σ-radical cation with the electron hole in the central σ-bond.

## Structural Analysis by X-ray and Micro-ED

**Figure 2:**
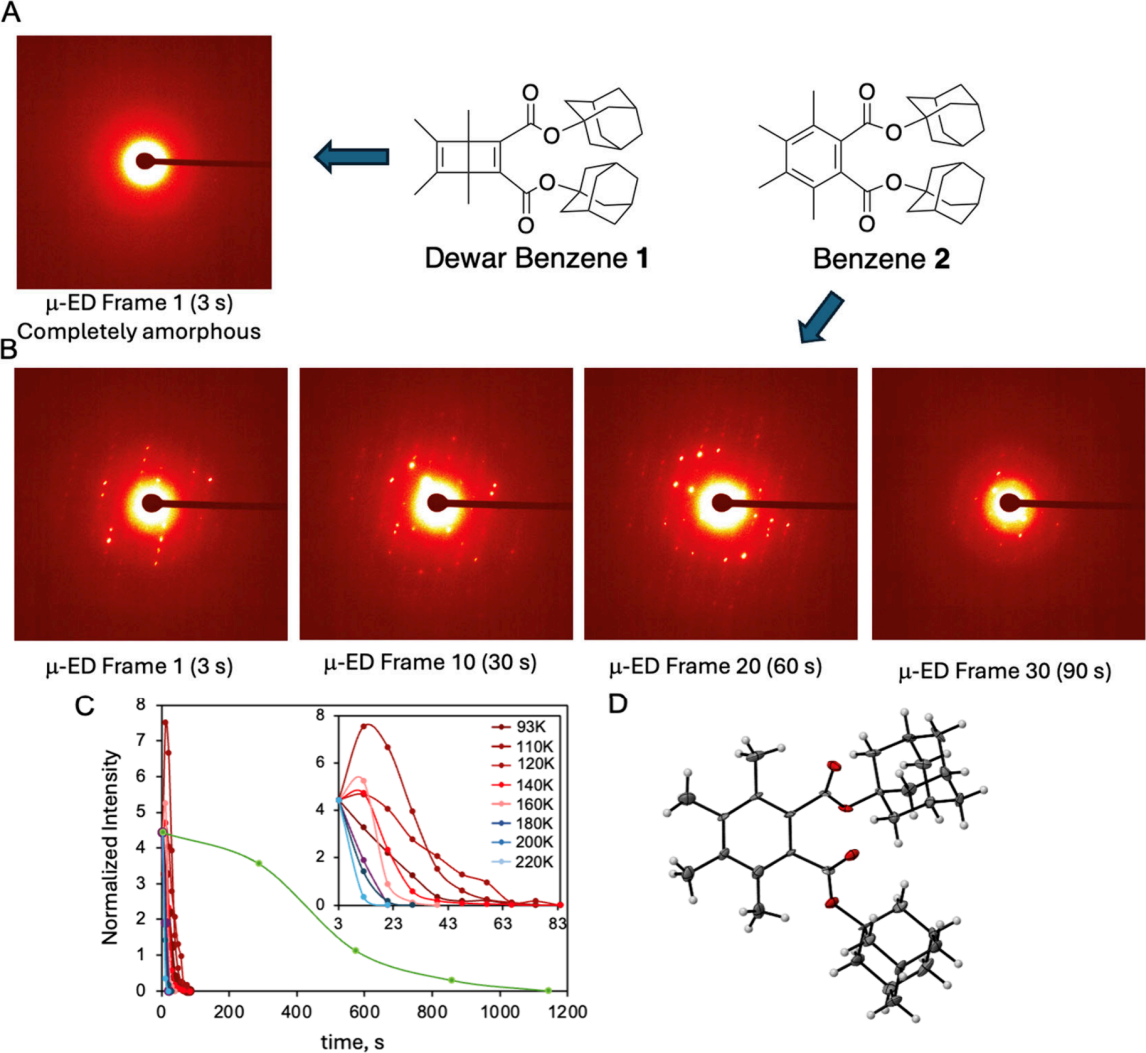
**Micro-ED (μ-ED) analysis of crystals of 1 and 2**. (A) Crystals of Dewar benzene **1** exposed to the electron beam at 298 K amorphized within the 3 s that it takes to acquire the first image, as indicated by the lack of diffraction spots. (B) The loss of crystallinity of benzene **2** at 298 K occurs over much longer time periods, up to 90 s. (C) Plot of diffraction intensity vs time for Dewar benzene **1** as a function of temperature between 95 and 222 K bunched together in the first 100 s, and with expanded time axis in the inset. The green line corresponds to the decay for benzene **2** at 100 K over a time scale of several hundred seconds. The intensity was derived by integrating the area from arbitrary chosen low angle diffraction peaks. A detailed discussion is included in SI section 8.3.1. (D) Molecular structures of benzene **2** determined from μED data acquired at 100 K (see SI page S20 for experimental details).

X-ray diffraction of plate-like single crystals of **1** revealed a structure with two molecules per asymmetric unit (Figure 1D). Crystals of **1** pack with segregated chains of adamantyl groups and Dewar benzenes (Figure 1E), with nested Dewar benzenes potentially facilitating propagation of the electron transfer step (Figure 1F). Benzene **2** forms small crystals in the range of 1 to 10 μm and a thickness of ca. 100 nm, making them good candidates for micro-ED experiments. Notably, our hypothesis suggests that micro-ED experiments on crystals of **1** and **2** would have different degradation rates despite having the same chemical composition. While part of the electron energy deposited in **1** should be funneled to the chain reaction, decomposition of **2** should be chaotic. Accordingly, data acquisition at 298 K using a 300 keV electron beam with an electron flux density of 0.027 e^–^/Å^2^ s resulted in the loss of diffraction signals of Dewar benzene **1** within the first 3 s needed to collect the first diffraction image (Figure 2A). By contrast, diffraction from microcrystals of **2** acquired under the same conditions decayed over 90 s (Figure 2B). While diffraction spots of **1** measured as a function of temperature between 220 and 93 K decayed to baseline within 10 and 60 s, potentially due to sample amorphization as the result of product accumulation, those from crystals of **2** measured at 100 K persisted for a few hundred seconds (Figure 2C). Diffraction data collected from five crystals of **2** at 100 K (Figure 2D) was solved in the space group *P*2_1_/*c* with one molecule per asymmetric unit. A detailed description of the data acquisition and structure determination of benzene **2** are described in the Supporting Information section (SI page S7).

## Single Electron Transfer (SET) Chain Reactions in Solution and in Crystals

**Figure 3:**
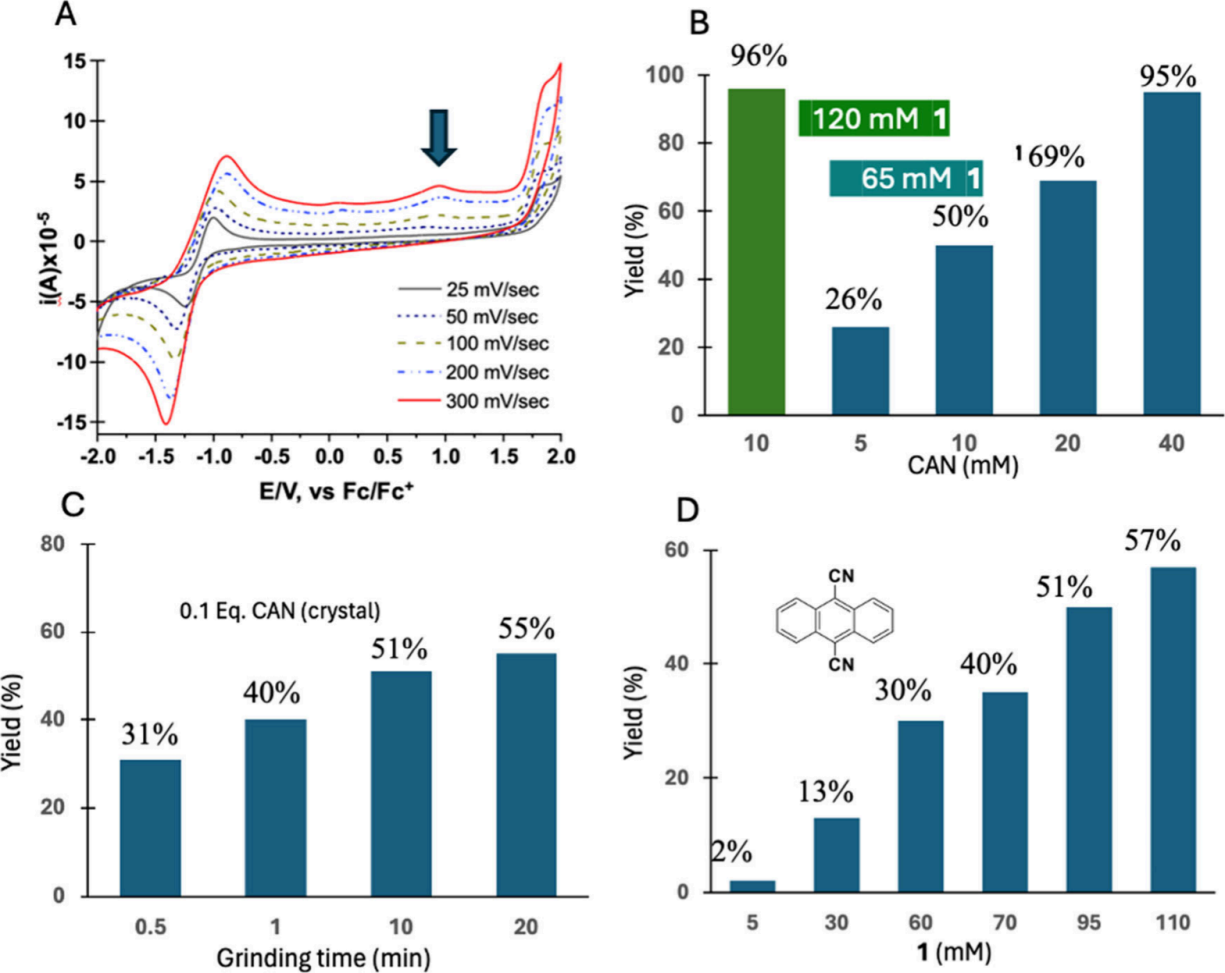
**Chain reaction of 1 in solution and in crystals with chemical and photochemical oxidants.** (A) Sequential cyclic voltammetry scans of 2 mM Dewar benzene **1** between −2.0 and 2.0 V vs Fc/Fc^+^ in acetonitrile solution as a function of scan rate between 25 mV/s and 300 mV/s. The shape of the curve is identical to the one obtained with benzene **2** except for the peak indicated by the arrow at ∼1 V, and the fact that current grows with increasing number of scans, suggesting an electrochemical reaction from **1** to **2**. (B) Reaction yield of the valence-bond isomerization reaction of **1** at 120 mM and 65 mM Dewar benzene **1** in acetonitrile solution initiated by substoichiometric ceric ammonium nitrate (CAN, *E*_Ce(IV)/Ce(III)_ = 0.97 vs Fc/Fc^+^) from 10 to 40 mM. (C) Mechanochemical reaction by grinding polycrystalline Dewar benzene **1** (10 mg) with 0.1 equiv of CAN as a function of time. (D) Reaction yield from the photoinduced chain reaction of 5 to 110 mM **1** using 0.25 mmol of 9,10-dicyano anthracene using λ = 420 nm.

Consistent with early reports, cyclic voltammetry measurements with **1** and **2** showed virtually identical oxidation potentials [(E (**1/1**^•+^) ≈ E (**2/2**^•+^) = 1.87 V vs Fc/Fc^+^]. However, a broad signal just above baseline was observed in the case of **1** between 0.7 and 1.2 V (Figure 3A, arrow) with an intensity that varied as a function of scan rate. This suggested that oxidation of **1** may occur at lower potentials with a chain reaction leading to the formation of **2** without producing current, until benzene **2** becomes oxidized at ca. 1.87 V. This was confirmed using ceric ammonium nitrate {CAN, (NH_4_)_2_[Ce(NO_3_)_6_]}, which has an oxidation potential of 1.21 vs Fc/Fc^+^ and can only be effective if the oxidation of **1** occurs below this value. Reaction of **1** with CAN occurred efficiently in solution (Figure 3B) and in mechanochemical solid-to-solid reactions (Figure 3C). Reactions carried out for 2 h at 298 K with 65 mM acetonitrile-benzene (1:20 v/v) solutions of **1** and CAN concentrations varying from 0.1 to 0.4 equiv led to yields from 25% to 95%, revealing the catalytic nature of CAN and the dependence of reaction yield on oxidant concentration. An increase of up to 96% in the yield of **2** as the concentration of **1** changed from 65 mM to 120 mM is consistent with a chain reaction by diffusion-mediated hole transfer from **2**^•+^ to **1**. A novel solid-to-solid interfacial reaction using 10 mg of **1** ground with 10 mol % of CAN produced **2** in yields that varied from 20% to 55% as mechanical grinding times increased from 0.5 to 20 min, demonstrating a radical-ion-mediated chain reaction initiated at the solid–solid interface. A comparison of reactions carried out with 0.5 mL of a 65 mM solution (15.9 mg) or with 10 mg of a polycrystalline **1** using 10 mol % of CAN at 298 K for 2 h and 0.5 min, respectively, revealed reaction velocities that differ by a factor or 99.3 in favor of the solvent-free reaction (i.e., 2.26 × 10^–6^ mmol s^–1^ in solution and 2.11 × 10^–4^ mmol s^–1^ in the solid state). Finally, as shown in Figure 3D, photochemical generation of **1**^•+^ with 9,10-dicyano anthracene (DCA) as a SET sensitizer (E_DCA*/DCA•–_ > 2.0 V vs SCE) using λ > 420 nm gives reaction yields that depend on the initial concentration of **1** under conditions where the initiation step is identical, which is also indicative of a chain reaction.

## Microscope Electron Beam-Induced Chain Reaction

**Figure 4:**
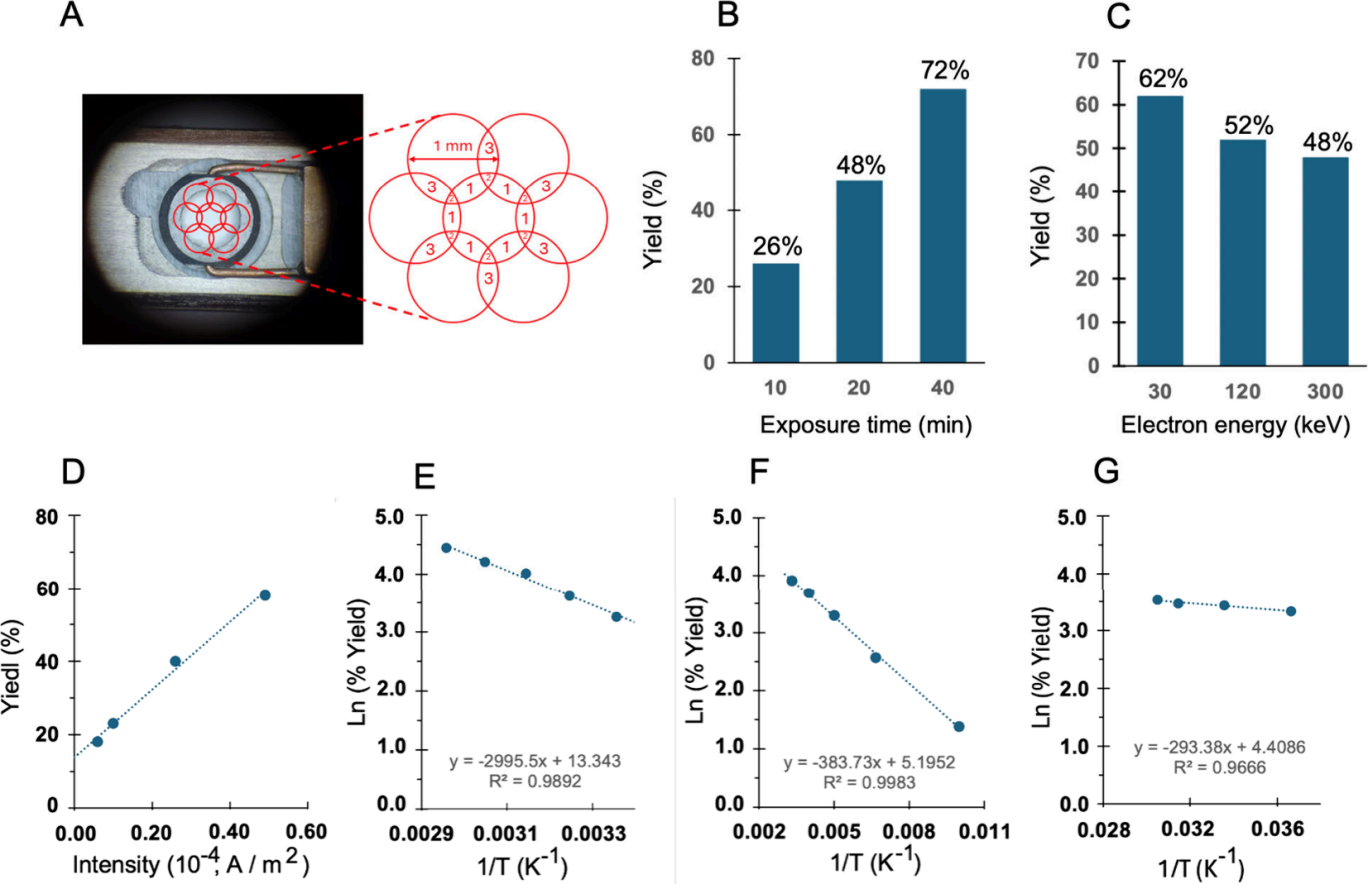
**Chain reaction of Dewar benzene 1 in the electron microscope.** (A) Electron microscope 2 mm diameter grid holding 0.2–0.3 mg of polycrystalline sample **1** indicating the positions of electron beam exposure, each covering a 1 mm surface area. (B) Reaction yield for the chain reaction from **1** to **2** under an electron beam energy of 300 keV and a flux density of 4 × 10^–6^ e^–^/Å^2^ s. (C) Yield of benzene 2 as a function of electron energy from 30 to 300 keV for 20 min and a flux density of 4 × 10^–6^ e^–^/Å^2^ s. (D) Yield of **2** upon exposure to different electron beams intensities (A = Amperes) for 5 min. (E) Arrhenius plot of **2** in acetonitrile solution using CAN as the oxidant gives an apparent activation energy of 6 kcal/mol. (F) Arrhenius plot of **2** under the electron beam between 300 and 100 K gives an apparent activation energy of 0.8 kcal/mol. (G) Arrhenius plot of the yield of **2** from a mechanochemical reaction between 273 and 320 K gives an apparent activation energy of 0.6 kcal/mol.

Electron beam irradiation experiments were conducted on a Thermo Fisher Spectra 300C TEM. Polycrystalline samples were deposited on Ted Pella 300 mesh copper grids with carbon/Formvar substrate and pressed with a flat metal piston to form a homogeneous thick film with ca. 0.2 mg of polycrystalline Dewar benzene **1** covering the 2 mm diameter grid (Figure 4A) with a thickness of ca. 20 μm. Using a value of 0.03 Å for the Binary-Encounter-Bethe (BEB) model for the electron impact ionization cross section of benzene at 300 keV,^15,16^ we estimate a total electron ionization mean free path of 2.3 μm, and up to 99% ionization for 10.4 μm thick samples. Experimental measurements confirmed that our 20 μm pellets are “opaque” to the transmission of electrons. Experiments were carried out with polycrystalline **1** using electron energies of 300 keV and a flux density of ca. 4 × 10^–6^ e^–^/Å^2^ s. As the electron beam has a smaller cross section than the pellet, the beam was displaced over the seven positions, with each position exposed to the incoming electrons by the same length of time. Effective beam exposure was subject to geometric corrections as described in the Supporting Information section (SI page S9). Exposure times of 10, 20, and 40 min resulted in conversion values of 26, 48 and 72% yield of benzene **2** as the only detectable product determined by ^1^H NMR analysis (Figure 4B). Knowing that the mean free path of the electron beam is inversely proportional to its kinetic energy, we carried out experiments with the same dose rate but beam energies varying by 1 order of magnitude. A modest change in product yields of 62, 52 and 48% for electron energies of 30, 120, and 300 keV, respectively, is qualitatively consistent with the modest increase in ionization cross section calculated by the BEB model (15). To test the role of secondary electrons we varied the intensity of the electron beam with the expectation that ionization by secondary electrons would lead to a nonlinear dependence of the product yield. However, experiments carried out at 300 keV with flux densities covering a factor of 30 between 3.7 × 10^–6^ to 1.3 × 10^–4^ e^–^/Å^2^ s showed a linear dependence (Figure 4D), suggesting that secondary electrons do not play a significant role.

## Temperature Dependence

The activation energy for a chain reaction, *E*_a_, can be approximated in terms of the relative activation energies for the initiation (*E*_i_), propagation (*E*_p_), and termination (*E*_t_) steps, as shown in eq 1:^17^
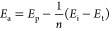
1where *n* is the order of the termination step with respect to the chain carrier. If the chain reaction has vanishing activation energies for initiation and termination, the apparent activation energy for product formation corresponds to the propagation step. Experiments carried out with the electron beam at 300 keV using a flux density of 3.8 × 10^–6^ e^–^/Å^2^ s produced **2** in yields varying from 4% to 50% over the range of 100 to 300 K, leading to an apparent activation energy of merely 0.8 kcal/mol in the solid state (Figure 4F). Similarly, mechanochemical reactions between **1** and solid CAN for 30 s at 275, 298, 318, and 328 K produced **2** in yields of 28, 31, 32 and 34%, respectively, which reflect an apparent activation energy of 0.6 kcal/mol, in excellent agreement with a solid-state chain reaction where ionization and propagation are remarkably favorable.

## Chain Reaction Length

**Figure 5:**
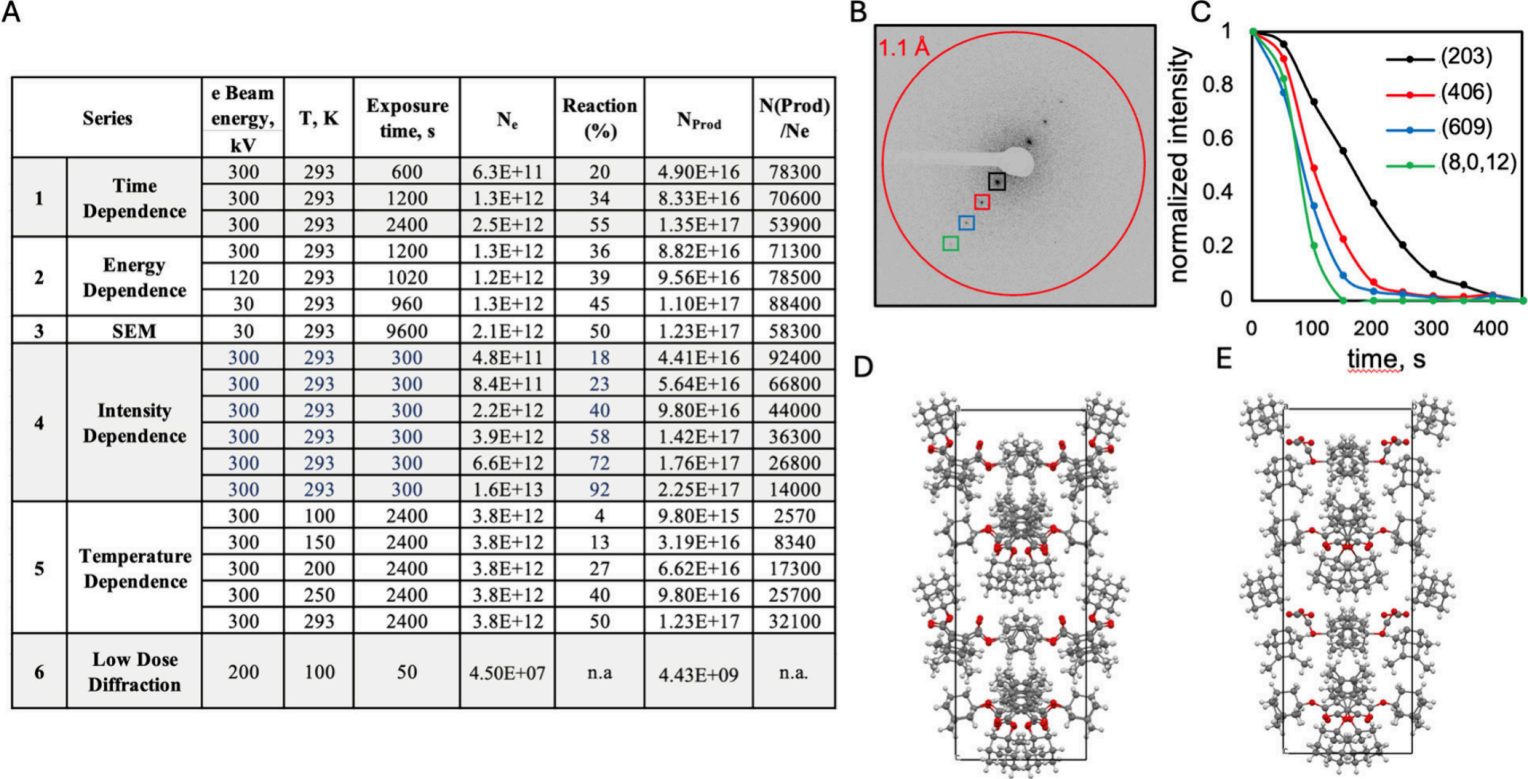
**Reaction chain length and micro-ED analysis in crystals of Dewar benzene 1.** (A) Summary of experiments with key variables indicating the number of rearrangements per incident electron for several experiment series. Chain lengths approach values of 90,000 per incident electron at low exposure and conversion values. The number of incident electrons (4.5 × 10^7^) and up to 90,000 reactions per electron suggest that all starting material (ca. 4.43 × 10^9^ molecules) was consumed after 50 s in the low dose single crystal diffraction experiment in set No. 6. (B) Diffraction image illustrating the [203] row line used to monitor the decay in intensity shown in (C), where faster decay of larger 2Θ indicates the rapid loss of long-range order. A comparison of (D) the packing structure obtained of **1** from single crystal X-ray diffraction and (E) the one obtained by optimized low exposure micro-ED.

The number of reactions per incident electron can be estimated from the number of electrons deposited in polycrystalline **1** and the number of product molecules formed. For the experiments in Figure 4B we used a flux density of 3.73 × 10^–6^ e^–^/Å^2^ s in an irradiated sample area *S* = 2.8 × 10^14^ Å^2^, such that the total electron flux for the exposed area becomes 1.04 × 10^9^ e^–^/s. Exposure times of 600, 1200, and 2400 s led to reaction yields of 26, 48 and 72%, respectively, corresponding to efficiencies of ca. 78,300, 70,560, and 53,900 product molecules per incident electron. These values are included in set (1) of Figure 5A along with those calculated from experiments carried out (2) as a function of electron energy (Figure 4C), (3) with low energy electrons in SEM mode, (4) as a function of beam intensity (Figure 4D), (5) as a function of temperature (Figure 4E), and (6) in a low dose micro-ED acquisition described below. The internal consistency among a large set of experiments is noteworthy, with reaction yields approaching 90,000 reaction per incident electron at low conversion values at 298 K. Variations in reaction yield as a function of electron beam energy and temperature are relatively weak and variations as a function of electron beam intensity are linear. Considering that incident electrons lead to the formation of X-rays, secondary electrons, backscattering events, charge trapping, atomic and molecular knockoff, as well as sample heating, the number of ionization events per incident electron should be relatively small, such that the number of reactions per ionization may be significantly higher.

## MicroED of Reactive Crystals of Dewar Benzene **1**

It was of interest to determine whether state of the art diffraction methods may be used to determine the structure of Dewar benzene **1**. To minimize damage during data collection we reduced the electron flux density 10-fold as compared with standard conditions, to ca. 0.003 e^–^/Å^2^ s, and used faster data collection with an exposure time of 0.5 s/frame and a tilt speed of 2 deg/s, as well as an Apollo direct electron detector. This translates to 1 frame/deg. Each data set was collected for crystals rotated from −50° to 50° (100 frames in total) and measurements were performed at 100 K with an electron energy of 200 keV. A multiple pass experiment with 10 full data collection scans using one crystal showed the loss of crystallinity within the [203] row to occur faster for high resolution spots (Figure 5B,C), with diffraction data retained up to ca. 1.25 Å resolution in a single pass. Structure analysis carried out by merging the best three data sets out of 20 showed that the structure cannot be solved using direct methods, intrinsic phasing, dual space, and charge flipping. An approximate model of the crystal structure can be obtained by the isomorphous replacement method using the X-ray (Figure 2D) structure with a result that has reasonable refinement statistics for microED data (e.g., R1 = 23.45%, GOF = 0.939) and compares well with the X-ray structure (Figure 5D,E). It is rather remarkable that low dose microED data collection can provide such a level of information for Dewar benzene **1** despite its high chemical vulnerability to the electron beam. While these results underscore some of the challenges associated with microED crystal structure determination of molecules primed to react upon electron beam exposure, they also highlight the remarkable development of technological and experimental strategies that continue to expand and improve this powerful structural elucidation method.

## Conclusions

The structural damage caused by the 200–300 keV beams of electron microscopes on molecular crystals can be funneled into clean chemical reactions using probes designed to undergo radical cation-mediated chain reactions. Evidence for a radical cation chain reaction of Dewar benzene **1** to benzene **2** was obtained using ceric ammonium nitrate (CAN) and 9,10-dicyanoanthracene, respectively, as thermal and photochemical single electron transfer oxidants. Mechanochemical experiments with polycrystalline **1** and substoichiometric CAN (e.g., 0.1 equiv) confirmed that the chain reaction occurs in the solid state, and electron beam-initiated reactions as a function of time, beam intensity, and beam energy, reveal chain reactions with as many as ca. 90,000 product molecules formed per incident electron. A weak temperature dependence of the e-beam reaction revealed an activation energy of ca. 0.8 kcal/mol, consistent with a value of 0.6 kcal/mol determined for the mechanochemical reaction between **1** and CAN. Micro-ED analysis of **1** and **2** revealed extreme vulnerability differences for samples with the same chemical composition. Altogether, these results show that well-behaved reactions under the flux of an electron beam from a cryo-electron microscope are possible and open a window for a better understanding of the various dissipation pathways that could lead to the mitigation of electron beam degradation mechanisms and to leveraging the electron microscope as a forge for chemical syntheses in the solid state.

## Data Availability

Crystallographic information files CCDC 2352506 and 2353011 contain crystallographic data for compounds **1** (X-ray) and **2** (μ-ED). These data can be obtained free of charge from The Cambridge Crystallographic Data Centre via www.ccdc.cam.ac.uk/structures.

## Supporting Information Available

Synthesis and characterization of compounds **1** and **2**; electrochemical analysis; chemical and photosensitized single electron transfer reactions; X-ray diffraction analysis of crystalline **1** and electron diffraction analysis of **1** and **2**; beam ionization experiments under various conditions (PDF)
Structural data for **1** and **2** (ZIP)
