# Supplementary material for: From Beam Damage to Massive Reaction Amplification under the Electron Microscope: An Ionization-Induced Chain Reaction in Crystals of a Dewar Benzene
Source: ACS Cent Sci. 2024 Dec 6;10(12):2346–52. doi: 10.1021/acscentsci.4c01429 (PMC11672530; doi:10.1021/acscentsci.4c01429)
Supplement: Supplementary file 1 — oc4c01429_si_001.pdf [file oc4c01429_si_001.pdf]

Supporting Information

From Beam Damage to Massive Reaction Amplification Under the Electron Microscope: An Ionization-Induced Chain Reaction in Crystals of a Dewar Benzene

Krzysztof A. Konieczny†[1,2], Indrajit Paul†[1], Jose A. Rodriguez[1], Miguel A. Garcia-Garibay[1]*

[1]Department of Chemistry and Biochemistry, University of California at Los Angeles; Los Angeles, CA 90095, United States

[2]Faculty of Chemistry, Wrocław University of Science and Technology, Wybrzeże Wyspiańskiego 27, Wroclaw 50-370, Poland

mgg@chem.ucla.edu

† These authors contributed equally to this work

TABLE OF CONTENTS.

1. Material and Methods……………………………………………………..….......................S3
2. Synthetic Procedures………………………………………………………….……………...S4-S5
 2.2 Synthesis and Characterization of Dewar Benzene **1**……………….…….……………………S4
 2.3 Synthesis and Characterization of Benzene **2**…....………………………….………………..….S5
3. Single electron transfer chain reactions in solution and in crystal using Ceric Ammonium Nitrate {CAN: $(NH_4)_2[Ce(NO_3)_6]$} as an oxidant at 25°C......…………..……………………………S5-S6
 3.2 In solution……………………………………………………………………………...….S5
 3.3 In crystal.……………………………………………………………… ………..…....S6
4. Electron transfer chain reaction in solution using CAN at different temperatures……….……S6
5. Electron transfer chain reaction in crystal using CAN at different temperatures………………...S6
6. Photochemical Isomerization of Dewar Benzene **1** to Benzene **2** with 9,10-dicyano anthracene (**DCA**) as a single electron transfer (SET) sensitizer……………………………………………S7
7. X-ray diffraction………………………………………………………………………………S7-S8
8. Electron Beam experiments………………………………………………………………....S8-S24
 8.2 Instruments……………………………………………………………….……………S8-S9
 8.3 Electron Beam irradiations…………………………………………………….…………...S9-S14

8.3.1 General preparation procedure and geometry corrections…………….……….……….…..S9-S11
8.3.2 Example calculations and error analysis……………………………….….…………...S12-S13
8.3.3 Chain Reaction Under Scanning Electron Microscopy (SEM)………………………….S14
8.4 Electron Diffraction…………………………………………………………..………..S14-S25
8.4.1 Diffraction properties examination…………………………………….…………S14-S17
8.4.2 Low Dose MicroED for Dewar Benzene **1**…………………...……….………S17-S20
8.4.3 MicroED crystal structure of recrystallized Benzene **2**……….……….…………S20-S25
8.4.4 Structures comparison…………………………………………………….……S25-S26
9. NMR…………………………………………………………………………………S26-S39
10. Electrochemistry……………………………………………………………………...S40-S41
11. References……………………………………………………………………….S41-S42

1. Materials and Methods

All solvents were dried by distillation prior to use whereas all commercially obtained reagents were used as received unless otherwise specified. (3s,5s,7s)-adamantan-1-ol was purchased from Sigma Aldrich, EDC was purchased from Oakwood chemicals, DMAP was purchased from AVOCADO privet limited. **DB-CO_2H** was synthesized using known literature procedure.[1] ^{1}H and ^{13}C NMR spectra were recorded on Bruker Avance (400 MHz & 500 MHz) spectrometers using a deuterated solvent as the lock and residual protiated solvent as internal reference ($CDCl_3$: δ_H 7.26 ppm, δ_C 77.0 ppm; C_6D_6: δ_H 7.15 ppm, δ_C 128.36 ppm). The following abbreviations were used to define NMR peak patterns: s = singlet, d = doublet, t = triplet, brs = broad signal, m = multiplet. The coupling constants are given in Hertz (Hz) and, wherever possible, assignment of protons is made. The carbons in the molecular skeletons were not necessarily numbered following the IUPAC nomenclature rules; numeration was exclusively done for assigning NMR signals. High-resolution mass spectrum data were recorded on a DART spectrometer in positive (ESI+) and negative (ESI-) ion mode. Melting point values were recorded on a Melt-Point II® apparatus. Infrared spectra were recorded on a PerkinElmer® Spectrum Two spectrometer equipped with a universal ATR sampling accessory. Infrared spectra were recorded on a Perkin-Elmer 1000 Series FT-IR spectrometer. Column chromatography was performed using silica gel (Millipore, 60 Å, 0.0063-200 mm, 70-230 mesh) as stationary phase. Merck silica gel (60 F254) or neutral alumina (150 F254) sheets were used for thin layer chromatography (TLC). Electrochemistry was performed using either a Gamry Instruments Interface 1000-E or a CH Instruments 760E potentiostat. Electron Beam experiments including diffraction, imaging and irradiation were conducted on Thermo Fisher Spectra 300C TEM (Transmission Electron Microscope) equipped with Ceta-D camera for diffraction pattern acquisition Scanning Electron Microscopy (SEM) experiment was performed on ZEISS Supra 40VP SEM.

2. Synthetic Procedure

2.1 Synthesis of Dewar benzene 3,4,5,6-tetramethyl-1,2-diadamantyl-dicarboxylate (1)

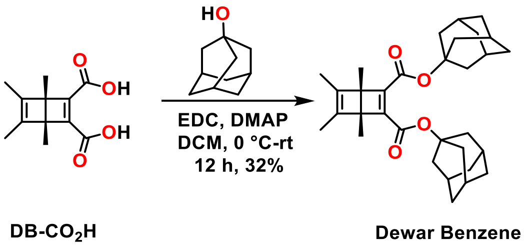

Scheme S1. Synthetic scheme for Dewar benzene 3,4,5,6-tetramethyl-1,2-diadamantyl-dicarboxylate (**1**).

A 25 mL 3-neck flask round bottom flask was flame dried and charged with compound **DB-CO_2H**[7] (100 mg, 441 µmol, 1.00 equiv), (3s,5s,7s)-adamantan-1-ol (201 mg, 1.32 mmol, 3.00 equiv), DMAP (38.0 mg, 309 µmol, 0.70 equiv,). This was followed by the addition of 5 mL anhydrous DCM and EDC (211 mg, 1.10 mmol, 2.5 equiv) stirring at 0 °C. The reaction was allowed to stir for 1 hour at 0 °C and slowly warm it to 25 °C and continue stirring for another 12 hours. After completion of the reaction (TLC monitored), the reaction mixture was subjected to column chromatography without workup utilizing a gradient solvent system composed of hexanes: ethyl acetate (100:0, 50:1, 25:1, 20:1). The purified product was isolated as a white crystalline solid (70 mg, 441 µmol, 32% yield). R_f = 0.6 in hexanes: ethyl acetate (3:1). The compound was furthermore crystallized in *n*-hexane by slow solvent evaporation at room temperature for single crystal X-ray diffraction.

Mp: 155-157 °C. **IR**: 737, 813, 838, 883, 936, 967, 984, 996, 1055, 1084, 1098, 1169, 1221, 1264, 1294, 1326, 1254, 1369, 1440, 1456, 1630, 1698, 1722, 2854, 2910 cm^{-1}. **^{1}H NMR (500 MHz, benzene-*d*$_6$)** δ = 2.34-2.29 (m, 12H), 1.98-1.90 (m, 6H), 1.66 (s, 6H), 1.55-1.46 (m, 6H), 1.46-1.37 (m, 6H), 1.34 (s, 6H) ppm. **^{13}C NMR (DMSO-*d*$_6$, 125 MHz):** δ = 161.8, 152.1, 144.1, 81.0, 56.6, 42.1, 36.6, 31.4, 11.49, 10.5 ppm. **HRMS (DART)** calcd for $C_{32}H_{42}O_4$ $[M+H]^+$ = 491.31613; found 491.31638

2.2 Synthesis of benzene 3,4,5,6-tetramethyl-1,2-diadamantyl-dicarboxylate (2)

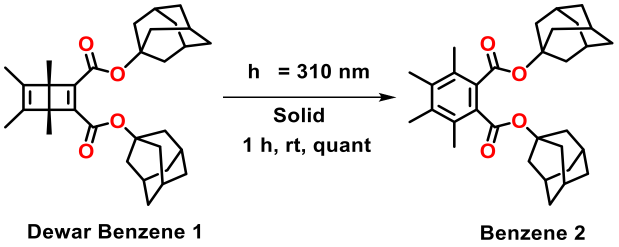

A well-ground microcrystalline powder sample of Dewar benzene **1** (**DB-CO$_2$Ad**) (10 mg, 20.3 μmol) was placed between two glass plate and irradiated in Hanovia (Hg lamp) for 60 min using 310 nm filter. Then the solid powder was taken directly for spectroscopic analysis. The compound was recrystallized from hexane for micro-electron diffraction purposes.

Yield: quantitative. **Mp**: 177 °C. **IR**: 738, 813, 853, 896, 938, 1055, 1184, 1205, 1284, 1322, 1355, 1456, 1573, 1716, 2852, 2909 cm^{-1}. **^{1}H NMR (500 MHz, benzene-*d*$_6$)** δ = 2.50-2.42 (m, 12H), 2.27 (s, 6H), 2.06-1.99 (m, 6H), 1.84 (s, 6H), 1.63-1.53 (m, 6H), 1.52-1.42 (m, 6H) ppm. **^{13}C NMR (DMSO-*d*$_6$, 125 MHz):** *δ*= 168.8, 136.7, 132.6, 130.7, 81.7, 42.1, 36.7, 31.5, 17.9, 16.4 ppm. **HRMS (DART)** calcd for $C_{32}H_{42}O_4$ [M+H]$^+$ = 491.31613; found 491.31622

3. Single electron transfer chain reactions in solution and in crystal using Ceric Ammonium Nitrate {CAN: $(NH_4)_2[Ce(NO_3)_6]$} as an oxidant at 25 °C.

3.1 <u>In Solution (65 mM):</u>

Dewar benzene (**1**) (16.0 mg, 32.6 μmol, 1 equiv) was taken in a 2 mL oven dried vial and dissolved in 480 μL benzene-*d*$_6$. Thereafter Ceric Ammonium nitrate (CAN) (5-40 mol%) in 24 μL acetonitrile was added in one portion and stirred the mixture for 2 h at 25 °C. The progress of the reaction was monitored by ^{1}H NMR using small aliquot of the reaction mixture in benzene-*d*$_6$. NMR yield of benzene **2** was determined by relative integration of product and starting material signals.

In Solution (120 mM):

Dewar benzene (**1**) (24.7 mg, 50.4 µmol, 1 equiv) was taken in a 2 mL oven dried vial and dissolved in 400 µL benzene-d_6. Thereafter Ceric Ammonium nitrate (CAN) (2.76 mg, 5.04 mmol, 0.1 equiv) in 20 µL acetonitrile was added in one portion and stirred the mixture for 2 h at 25 °C. The ^{1}H NMR was taken using small aliquot of the reaction mixture in Benzene-d_6. NMR yield of benzene **2** was determined through relative integration.

3.2 In Crystal:

Dewar benzene **1** (10.0 mg, 20.4 µmol, 1.00 equiv) was taken in oven dried mortar and Ceric Ammonium nitrate (CAN) (1.11 mg, 2.04 mmol, 0.10 equiv) was added in one portion. Afterwards, the mixture was grinded with a pestle for 0.5 to 20 minutes. Then the solid mixture was dissolved in benzene-d_6 and conducted ^{1}H NMR to determine the formation of benzene **2** by relative integration.

4. Electron transfer chain reaction in solution using CAN at different temperatures.

Oven dried six 2 mL vials were charged with Dewar benzene (**1**) (16.0 mg, 32.6 µmol, 1.00 equiv) and dissolved in 480 µL benzene-d_6. Thereafter, Ceric Ammonium nitrate (CAN) (893 µg, 1.63 mmol, 0.05 equiv) in 24 µL acetonitrile was added in one portion to the individual vials and stirred the mixture for 2 h at different temperatures (0 °C, 25 °C, 35 °C, 45 °C, 55 °C and 65 °C). The progress of the reaction was monitored though ^{1}H NMR using small aliquot of the reaction mixture in Benzene-d_6. NMR yield of benzene **2** was determined through relative integration.

5. Electron transfer chain reaction in crystal using CAN at different temperatures.

Dewar benzene **1** (10.0 mg, 20.4 µmol, 1.00 equiv) was taken in oven dried mortar and Ceric Ammonium nitrate (CAN) (1.11 mg, 2.04 mmol, 0.10 equiv) was added in one portion. Afterwards, the mixture was grinded with a pestle for 30 seconds at different temperatures (0 °C, 25 °C, 45 °C, 55 °C). Then the solid mixtures were dissolved in benzene-d_6 and conducted ^{1}H NMR to determine the benzene **2** formation through relative integration.

6. Photochemical Isomerization of Dewar benzene 1 to benzene 2 with 9,10-dicyano anthracene (DCA) as a single electron transfer (SET) sensitizer

Dewar benzene **1** (5, 30, 60, 70, 95 and 110 µmol) were taken in five different 2 mL oven dried vial and dissolved in thoroughly degassed 500 µL benzene-d_6. Thereafter 9,10- dicyanoanthracene (DCA) (0.25 mmol) in 25 µL acetonitrile was added in one portion to each vial of Dewar benzene **1** solutions and stirred the mixture for 30 min at 25 °C in Hanovia (Hg lamp) using 420 nm filter. The progress of the reaction was monitored though ^{1}H NMR using small aliquot of the reaction mixture in benzene-d_6. NMR yield of Benzene **2** was determined through relative integration.

7. X-ray diffraction

The crystals of dewar benzene **1** suitable for single-crystal X-ray diffraction experiment were obtained by recrystallization from hexane, leading to a single polymorph that melts with concomitant isomerization to benzene **2**. Single crystal data collection was performed at 100K on Bruker diffractometer with Apex-II CCD detector using sealed tube Mo-Kα radiation source. For data collection, reduction and absorption correction Apex2 software suit was used.[2] The structure was solved by direct methods in ShelXS[3] and refined by full-matrix least-squares on F^2 in ShelXL.[4] All non-hydrogen atoms were refined anisotropically and all hydrogen atoms were fixed in their corresponding positions with $U_{iso}(H)=1.5U_{eq}(C)$ for methyl groups and $U_{iso}(H)=1.2U_{eq}(C)$ for remaining hydrogen atoms. The two crystallographically independent molecules are characterized by having their Dewar benzenes orientationally disordered with alternating concave and convex faces having occupancies of 62% and 38% for one, and 43% and 57% for the other. While the adamantyl groups are not disordered, the atomic positions corresponding to the carboxylate groups are also duplicated in the same proportions. The equivalent bond lengths in disordered fragments only were restrained to a single value and refined as a single parameter. Selected crystallographic data are gathered in Tab. S1. The full data for this structure was deposited in CCDC with #2353011.[5] The unit cell and an asymmetric unit are presented in Fig. S1.

Table S1. Crystallographic data for Dewar benzene **1**.

Structure	Dewar benzene 1
Crystal system	Orthorhombic
Space group	$Pca2_1$
Z,Z'	8,2
a [Å]	12.662(4)
b [Å]	12.644(4)
c [Å]	33.789(10)
Volume [$Å^3$]	5410(3)
Reflections collected	85940
Reflections independent	16099
Reflections observed	12209
Completeness [%]	100
R_{int}	0.0905
R [$F^2>2\sigma(F^2)$], wR, S	0.0626, 0.1664, 1.039
$\Delta\rho_{max}$, $\Delta\rho_{min}$ [$eÅ^{-3}$]	0.303, -0.300

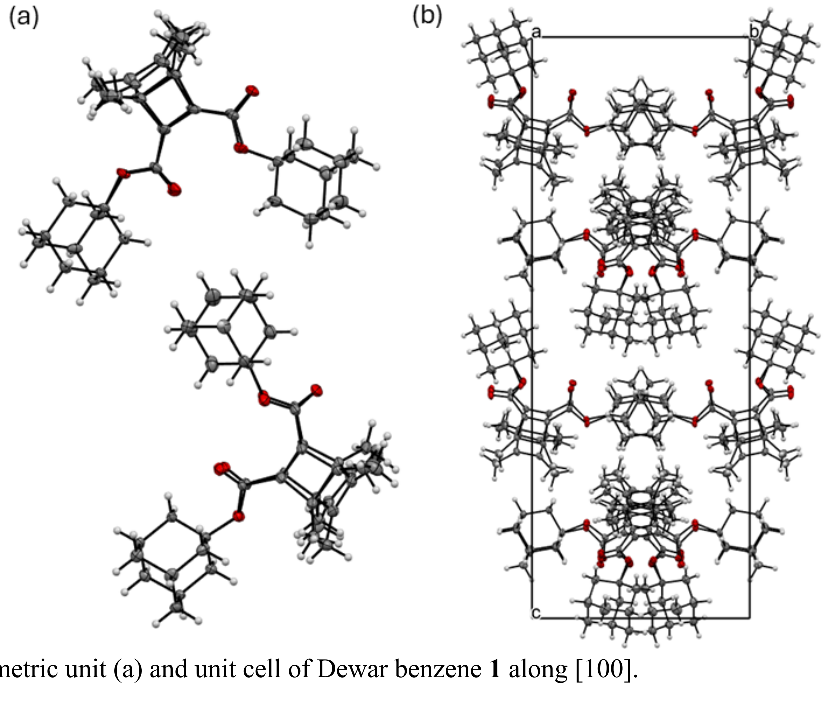

Figure S1. The asymmetric unit (a) and unit cell of Dewar benzene **1** along [100].

8. Electron Beam experiments

8.1 Instruments

Electron Beam experiments including diffraction, imaging and irradiation were conducted on Thermo Fisher Spectra 300C TEM (Transmission Electron Microscope) equipped with Ceta-D camera for diffraction pattern acquisition. The dose and associated quantities such as flux density,

fluence etc. were measured by direct current reading reported by the TEM for given beam diameter. For room temperature experiments a standard grid holder was used and for temperature dependent experiments Gatan 698 Elsa model. For all experiments Ted Pella 300 mesh copper grids with carbon/formvar substrate were used.

The exception from the above were low dose Electron Diffraction experiments which were performed on Talos F200C TEM with Apollo direct electron detector where 300 mesh gold grids with ultrathin carbon lacey were used.

8.2 Electron Beam irradiations

8.2.1 General preparation procedure and geometry corrections

Due to limit of quantification of NMR measurements it was necessary to ensure that proper amount of the sample was deposited on the TEM grid to correctly determine the Dewar benzene **1** to benzene **2** conversion. In this purpose a small pile of the crystalline compound was first placed on the TEM grid and then squeezed by a flat metal piston to obtain ca. 0.2 mg of the compound deposited on the grid in a form of an irregular disc of fairly homogenous thickness as it is presented in Fig. S2a.

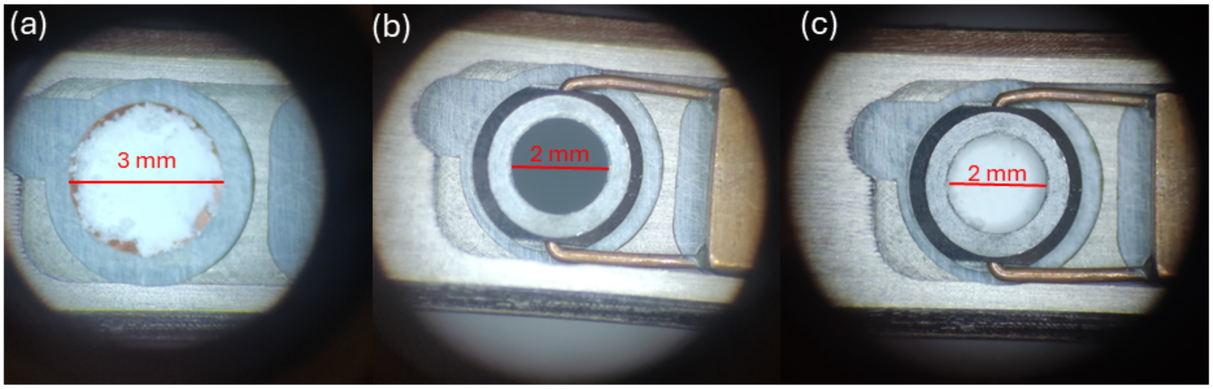

Figure S2. The grid with a sample prepared for EB irradiation experiment placed on the room temperature grid holder (a), empty grid holder with stabilizing ring on it (b) and the sample on a grid with a ring on it. (c).

The metal ring stabilizing the position of the grid overshadows part of the compound which is always excluded from direct electron exposure. Assuming equal compound distribution on the whole grid the total area of the compound on the grid is equal to 7.065 mm^2 and the area exposed to the electron beam equals 3.14 mm^2, what means that the total excluded area is 3.925mm^2 (55.5% of the total area). In practice however, most of the compounds that have contact with the metal ring stick to it since it has better adhesion to steel than to copper grid. Because of that we assume

that the metal ring overshadowing does not introduce conversion underestimation exceeding 10% because not exposed compound was in majority not used in NMR experiments.

The other factor is the side on which the grid was irradiated, i.e. either from top or the bottom of the grid, Fig. S3a and S3b. Most of the samples were irradiated from the top, however for all the samples irradiated through-the-grid the effective illuminated area was multiplied by 0.66, i.e. by the ratio between the red and yellow square in Fig. S3c.

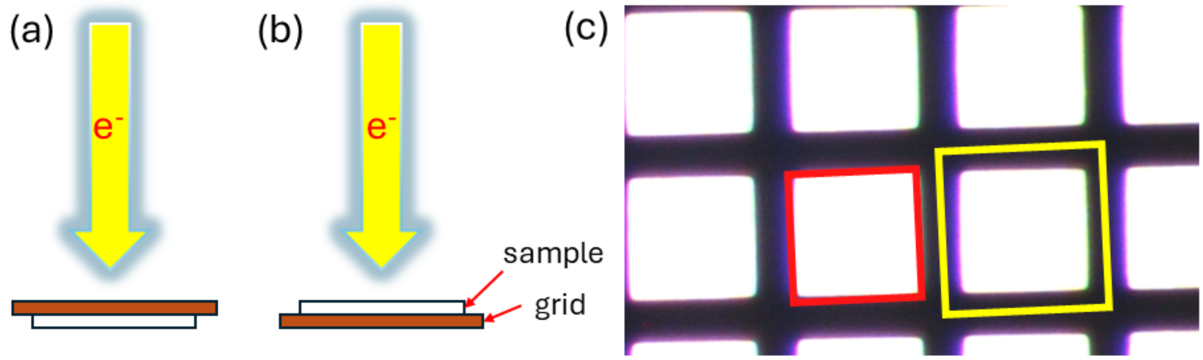

Figure S3. (a) Through-the-grid and (b) direct sample electron irradiation and (c) the geometrical representation of the illuminated (red square) and total (yellow square) area during through-the-grid irradiation.

The third factor was the geometry of the electron beam and its position relative to the grid. All the sample irradiations were conducted in TEM imaging mode for the beam expanded to 1 mm diameter. In order to irradiate the whole exposed area, after a given period of time the beam was moved to another region irradiated for the same amount of time until the whole grid was irradiated (7 irradiated circles per 1 grid in total), Fig. S4.

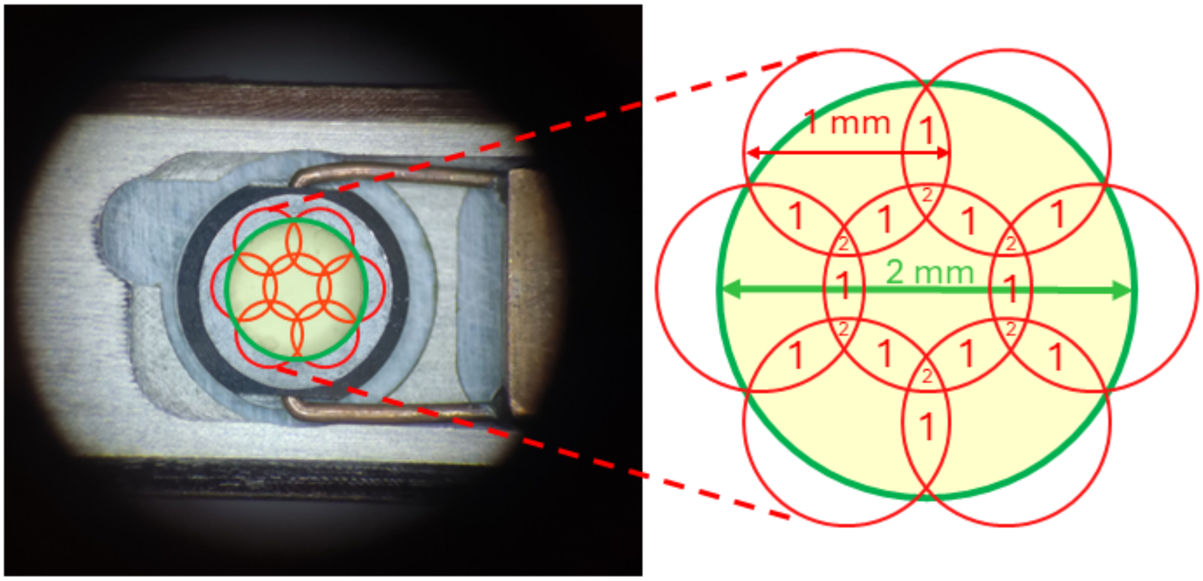

Figure S4. The beam-sample surface geometry. Areas marked as 1 and 3 were irradiated twice and areas marked as 2 were irradiated three times.

Since the amount of the compound on the grid was way too thick for electrons to penetrate it there was no TEM image to observe the exact position of the probe relatively to the sample, thus moving the beam was performed based on the relative position of the stage, what certainly introduced additional errors to the experiment. In some cases, certain degree of control was possible due to the discontinuities in the sample layer. Anyway, as it is shown in Fig S4. The effective irradiated area was bigger than 2 mm diameter circle area because certain regions overlapped twice, or three times thus were irradiated for longer period of time. This requires calculation of effective irradiated area in order to properly calculate the number of electrons interacting with the sample using the following equation:

$$A_{eff} = (A + 12(A_1) + 6A_2)G \approx 4.24\, mm^2 = 4.24 \cdot 10^{14} Å^2$$

Where,

$A = 3.14\, mm^2$- total irradiated area

G- 1 for directly irradiated samples and 0.66 for samples irradiated through the grid.

$A_1 \approx 0.07\, mm^2, \;\; A_2 \approx 0.016\, mm^2$- estimated areas marked as 1 and 2 in Fig. S4.

That effective irradiation calculations are valid for total number of electrons deposited on the sample calculations; however, the setup geometry introduces errors to all the measurements series due to e.g. overkill effect (for the regions irradiated 3 times the electrons might be still deposited even though the conversion already reached 100%).

All temperature dependent experiments were conducted in Elsa 698 cryotransfer holder, which geometry slightly differ from a standard room temperature holder, Fig, S5.

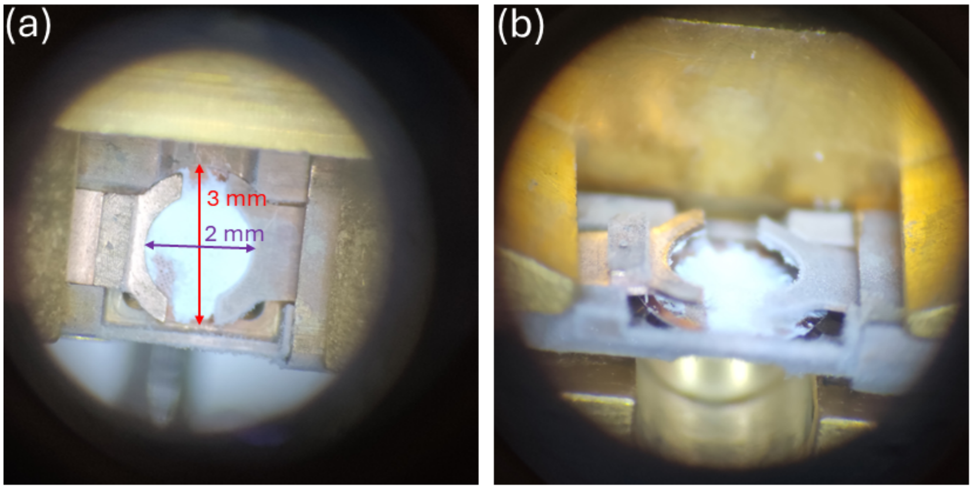

Figure S5. The grid mounted in Elsa 698 cryotransfer holder, (a) top view and (b) side view.

8.2.2 Example calculations and error analysis

Number of molecules calculation

$M_{w\,DB-Ad} = M_{w\,HB-Ad} = 490.84\ \frac{g}{mol}$ - molecular weight of the Dewar benzene 1 and benzene 2

$m \approx 0.2\ mg$ – approximate mass of the sample on the grid

$N_M \approx 2.46 \cdot 10^{17}$- approximate number of molecules on the grid

Exemplary number of electrons calculation

Flux density calculation, *D*:

$1A = 6.262 \cdot 10^{18} \frac{e^-}{s}$, $D = 5.95 \cdot 10^{-5} \frac{A}{m^2} = 3.726 \cdot 10^{14} \frac{e^-}{s \cdot m^2} = 3.726 \cdot 10^{-6} \frac{e^-}{s \cdot Å^2}$

Electron flux , F:

$$F = D \cdot A_{eff} = 3.726 \cdot 10^{-6} \frac{e^-}{s \cdot Å^2} \cdot 2.80 \cdot 10^{14} Å^2 = 1.04 \cdot 10^9 \frac{e^-}{s}$$

Total number of electrons (at 600 s):

$$N_e = F \cdot t = 1.04 \cdot 10^9 \frac{e^-}{s} \cdot 600\ s = 6.26 \cdot 10^{11} e^-$$

Number of molecules per electron:

$$N = \frac{N_M}{N_e} = \frac{2.46 \cdot 10^{17}}{6.26 \cdot 10^{11} e^-} \approx 400\ 000$$

Number of converted molecules per electron (at 20% conversion):

$$N_{conv} = N \cdot C = 400\,000 \ \cdot 0.2 \approx 80\,000$$

All calculations are gathered in Table S2

Table S2. Electron Beam Induced Dewar Benzene **1** to Benzene **2** Reaction Calculations.

Series type	EB energy, kV	T, K	m, g	Mw, g/mol	Nmol	flux density , A/m^2	flux density, e/s*Å^2	Illuminated area, Å^2	Corrected area, Å^2	Exposure time, s	flux, e/s	Nel	Nmol/Nel	Converted fraction	converted molecules/ electron
TEM time dependent	300	293	0.0002	490.84	2.45E+17	5.95E-05	3.73E-06	4.24E+14	2.80E+14	600	1.04E+09	6.26E+11	3.92E+05	0.20	7.83E+04
	300	293	0.0002	490.84	2.45E+17	5.95E-05	3.73E-06	4.24E+14	2.80E+14	1200	1.04E+09	1.25E+12	1.96E+05	0.36	7.06E+04
	300	293	0.0002	490.84	2.45E+17	5.95E-05	3.73E-06	4.24E+14	2.80E+14	2400	1.04E+09	2.50E+12	9.80E+04	0.55	5.39E+04
TEM Energy dependent	300	293	0.0002	490.84	2.45E+17	5.95E-05	3.73E-06	4.24E+14	2.80E+14	1200	1.04E+09	1.25E+12	1.96E+05	0.36	7.13E+04
	120	293	0.0002	490.84	2.45E+17	6.89E-05	4.31E-06	4.24E+14	2.80E+14	1020	1.21E+09	1.23E+12	1.99E+05	0.39	7.85E+04
	30	293	0.0002	490.84	2.45E+17	7.50E-05	4.70E-06	4.24E+14	2.80E+14	960	1.31E+09	1.26E+12	1.94E+05	0.45	8.84E+04
SEM	30	293	0.0002	490.84	2.45E+17	5.69E-06	3.56E-07	6.15E+14	6.15E+14	9600	2.19E+08	2.10E+12	1.17E+05	0.5	5.83E+04
TEM Intensity dependent	300	293	0.0002	490.84	2.45E+17	6.00E-05	3.76E-06	4.24E+14	4.24E+14	300	1.59E+09	4.78E+11	5.13E+05	0.18	9.24E+04
	300	293	0.0002	490.84	2.45E+17	1.06E-04	6.64E-06	4.24E+14	4.24E+14	300	2.81E+09	8.44E+11	2.91E+05	0.23	6.68E+04
	300	293	0.0002	490.84	2.45E+17	2.80E-04	1.75E-05	4.24E+14	4.24E+14	300	7.43E+09	2.23E+12	1.10E+05	0.4	4.40E+04
	300	293	0.0002	490.84	2.45E+17	4.92E-04	3.08E-05	4.24E+14	4.24E+14	300	1.31E+10	3.92E+12	6.26E+04	0.58	3.63E+04
	300	293	0.0002	490.84	2.45E+17	8.28E-04	5.18E-05	4.24E+14	4.24E+14	300	2.20E+10	6.60E+12	3.72E+04	0.72	2.68E+04
	300	293	0.0002	490.84	2.45E+17	2.02E-03	1.26E-04	4.24E+14	4.24E+14	300	5.36E+10	1.61E+13	1.52E+04	0.92	1.40E+04
TEM Temperature dependent	300	100	0.0002	490.84	2.45E+17	6.00E-05	3.76E-06	4.24E+14	4.24E+14	2400	1.59E+09	3.82E+12	6.42E+04	0.04	2.57E+03
	300	150	0.0002	490.84	2.45E+17	6.00E-05	3.76E-06	4.24E+14	4.24E+14	2400	1.59E+09	3.82E+12	6.42E+04	0.13	8.34E+03
	300	200	0.0002	490.84	2.45E+17	6.00E-05	3.76E-06	4.24E+14	4.24E+14	2400	1.59E+09	3.82E+12	6.42E+04	0.27	1.73E+04
	300	250	0.0002	490.84	2.45E+17	6.00E-05	3.76E-06	4.24E+14	4.24E+14	2400	1.59E+09	3.82E+12	6.42E+04	0.4	2.57E+04
	300	293	0.0002	490.84	2.45E+17	6.00E-05	3.76E-06	4.24E+14	4.24E+14	2400	1.59E+09	3.82E+12	6.42E+04	0.5	3.21E+04
TEM Low Dose on single microcrystal	200	100	3.62E-12	490.84	4.43E+09	4.79E-02	3.00E-03	3.00E+08	3.00E+08	50	9.00E+05	4.50E+07	9.84E+01	unknown	unknown

EB: electron beam, T: temperature, m: mass, Mw: molecular weight, Nmol: number of molecules, Nel: number of electrons

Systematic errors not included within corrections and error bars include the following:

1) Electron beam irradiation set-up geometry – overkill effect and overshadowing by the metal ring
2) Uncertainty of the exact position of the probe relatively to the sample
3) Mass loss due to the grid transfer to the holder and from the holder to the deuterated solvent solution.
4) Thickness homogeneity of the sample pellet on the grid

Main random errors include the following:

1) ^{1}H NMR measurement, +/- 5%
2) Mass measurement, +/- 5%

8.2.3 Chain Reaction under Scanning Electron Microscope Conditions

To confirm the reasonableness of the methodology used and the correctness of estimates and calculations as well as dose rate values readings an SEM (Scanning Electron Microscope) experiment was performed at 30 keV. The samples for SEM irradiation were prepared in the same way as for TEM experiments, i.e. 0.2 mg of the compound was deposited on the TEM grid (Fig. S1a) which was further mounted on the SEM stage using adhesive tape. The dose measurements were performed using Faraday cup. For the sample irradiation the electron beam was expanded to cover the whole grid containing the compound at once, thus no geometrical corrections were necessary. The total number of electrons deposited on the sample was adjusted to the TEM experiments by adjusting irradiation time to ensure approximately the same number of electrons per molecule. The conversion for 30kV, both from SEM and TEM was approximately the same what validates the correctness of dose rate measurements and geometrical corrections for TEM experiments.

8.3 Electron Diffraction

If not specified for all single tilt experiments the following parameters apply: Electron Beam (EB) accelerating voltage = 300 keV, exposure time=3 s/frame, tilt speed = 0.333 deg/s, beam diameter = 1.77 μm, flux density = 0.027 $e^-/Å^2s$.

8.3.1 Diffraction properties examination

At room temperature the Dewar benzene **1** to benzene **2** transformation is so fast that it is impossible to record any meaningful diffraction images using electron diffraction technique – the

first recorded diffraction image shows already complete sample amorphization. Even at low temperature the loss of crystallinity is way too fast to determine electron crystal structure for Dewar benzene **1**. The temperature dependent diffraction patterns for Dewar benzene **1** were collected as short datasets containing 3 frames per cycle only to ensure proper resolution of the plots. The data collection was repeated for each crystal at the given temperature as long as the diffraction peaks were observed (3-10 cycles). The exemplary diffraction images after the given electron beam exposure time for crystals measured at 110K and 220K are presented in Fig S6.

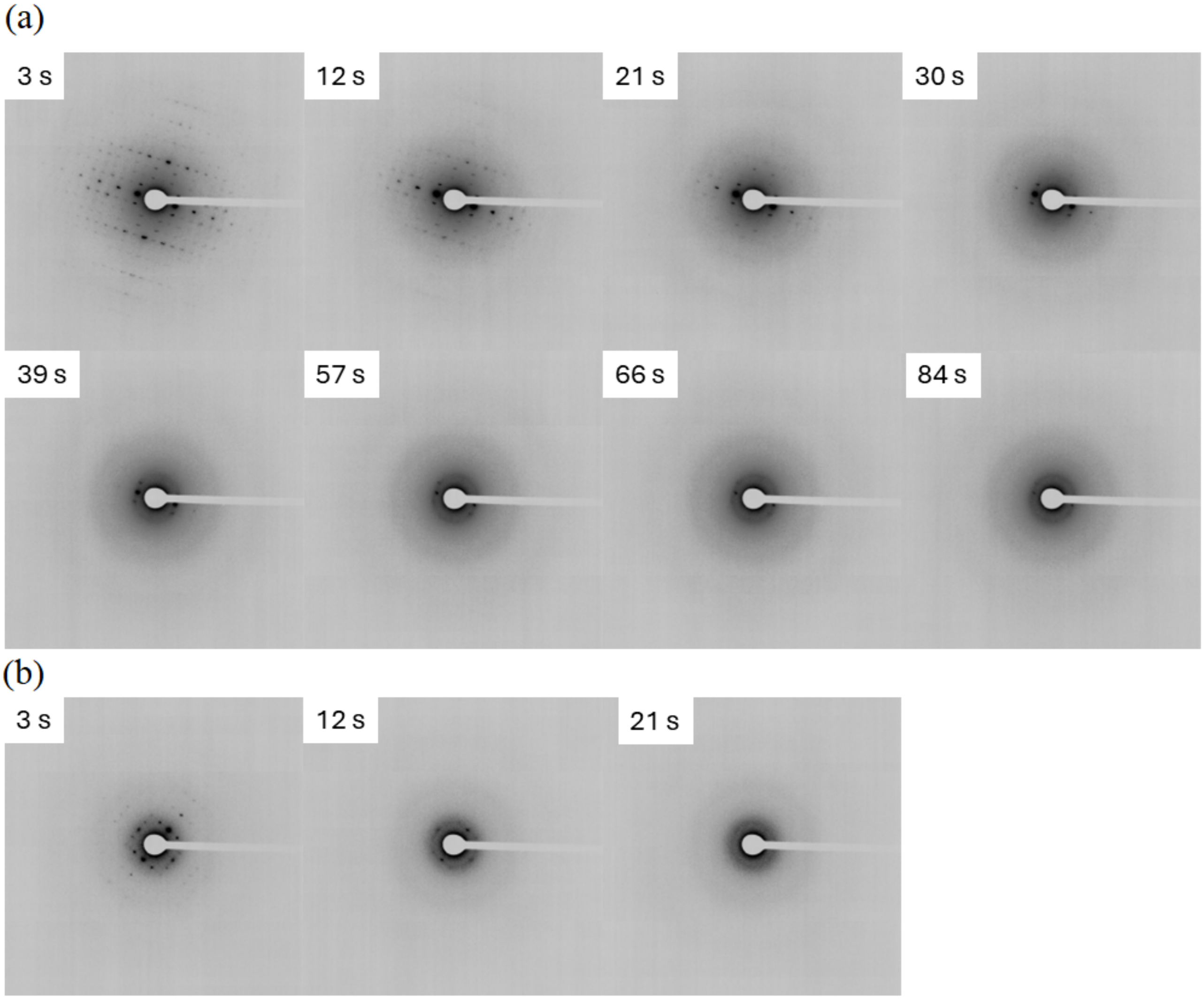

Figure S6. (a) μ-ED at 110K, (b) μ-ED at 220K

For each dataset the highest intensity low-angle diffraction peak area was integrated and intensity was plotted in a function of exposure time. To compare the peak intensity decrease at different temperatures the plots were normalized for background and rescaled relatively to the first recorded data point using the following equation:

$$I_{norm} = \left(\frac{I}{I_n} - 1\right) \cdot a$$

where,

I_{norm} – normalized intensity,

I – absolute intensity,

I_n – absolute intensity of the last datapoint representing background level,

a – scaling factor, individual for each dataset.

For comparison analogous experiment was performed for a microcrystal of benzene **2** at 100K to show the huge difference between the crystal damage induced by chemical reaction (Dewar benzene **1**) and radiation damage induced purely by the nature of organic matter interaction with high energy electrons, Fig. S7, top graph. For some Dewar benzene **1** plots at lower temperatures certain increase of the peak intensity is observed, what can give an impression of constructive crystal transformation, however this preliminary low-angle diffraction peak intensity increase is always accompanied by losing higher angle diffraction power (compare first two frames in Fig. S6a) which proves otherwise. It has to be kept in mind that each temperature data set was collected for different object, which varied in scope of initial quality and size (Fig. S7, right column), thus the crystal damage profile is not completely analogous for them. However regardless of this, there is a visible trend indicating that at lower temperatures the loss of crystallinity is slower (the time necessary for reaching the background is longer). From the images of Dewar benzene **1** microcrystals it can be noticed the rounding of the edges and corners implying sample amorphization.

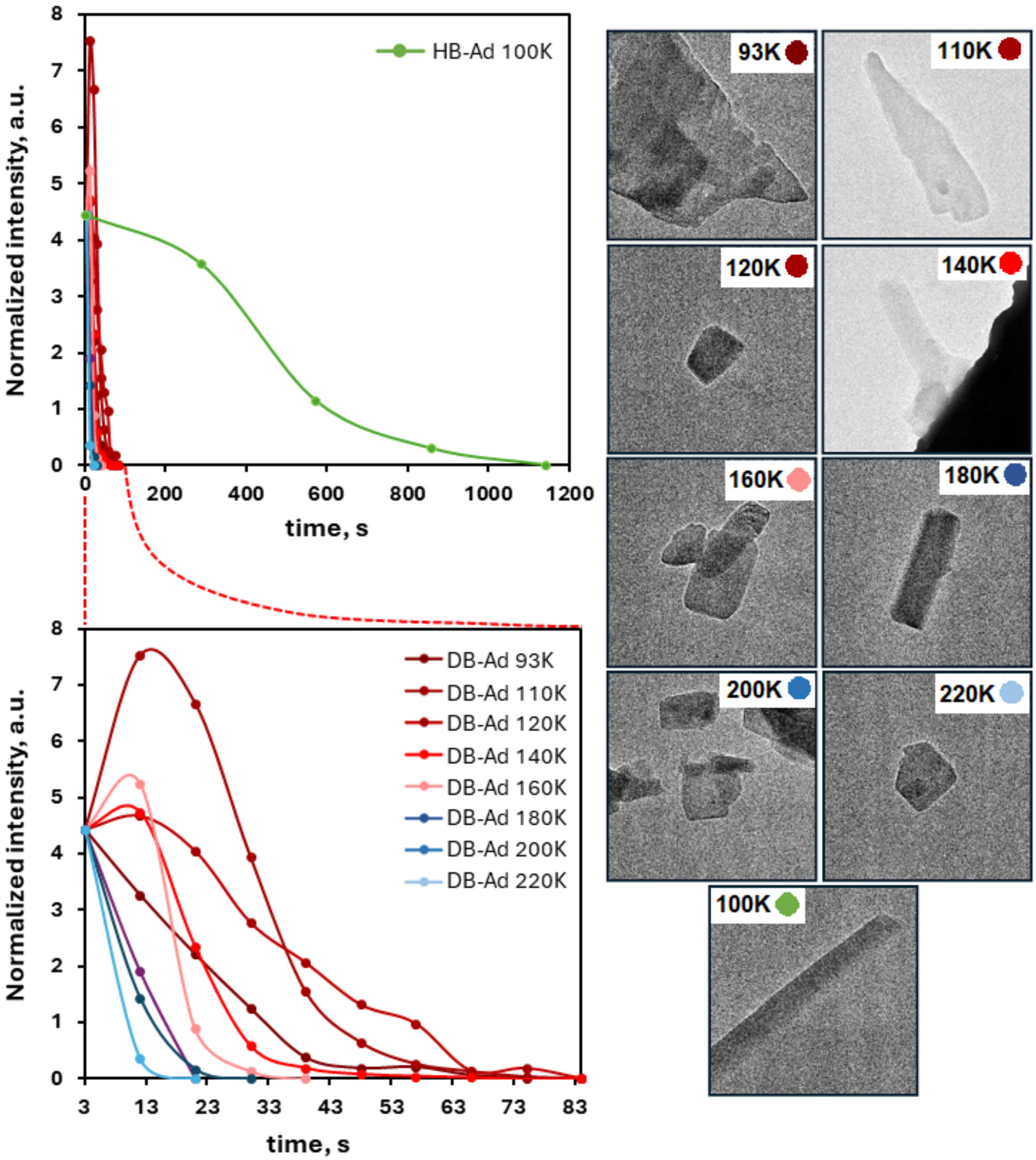

Figure S7. Low angle diffraction peak intensities normalized to a value of 4.5 a.u. exposure times of 3 s are a function of electron beam exposure time documented for Dewar Benzene **1** and Benzene **2**, and temperature dependence documented for Dewar Benzene **1** also shown in Fig. 2C in the main text were acquired from the crystals whose TEM images are shown on the right with color-coded labels to match the color of the curves.

8.3.2 Low Dose Micro-ED for Dewar Benzene 1

Data collection and reduction. Due to high rate of crystallinity loss the data collection for Dewar benzene 1 for micro-ED crystal structure determination purposes required special approach by reducing the electron beam dose approximately 10 times to ca. 0.003 $e^-/Å^2s$ and by faster data

collection using exposure time=0.5 s/frame and tilt speed = 2 deg/s, what translates to 1 frame per 1 deg. Each data set was collected for crystals rotated from -50 to 50 degrees (100 frames in total) and the measurements were performed at 100K using 200kV accelerating voltage.

For one crystal multiple pass experiment was performed (10 full data collection scans in total) to prove that the lower dose indeed prevents crystallinity loss. The decrease of reflection intensities within [203] row line in a function of electron beam exposure time is shown in Fig. S8 and the plot suggests that during the first dataset collection the crystallinity is remained, at least up to ca. 1.25Å resolution.

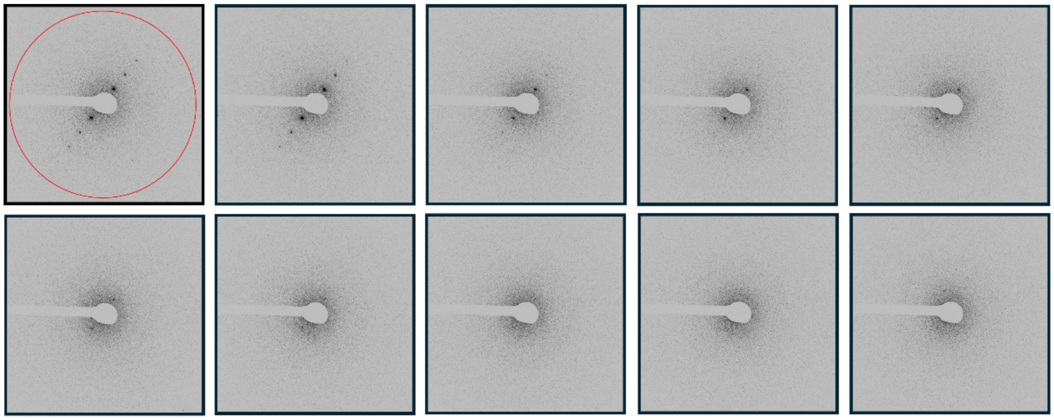

Figure S8. The decay of the reflections along [203] over electron beam exposure time. Each frame corresponds to one data collection cycle, i.e. one data point in Fig. 5C of the main text.

Based on the preliminary visual inspection of diffraction pattern collected for over 20 crystals 3 most promising datasets were chosen for data reduction in CrysalisPro software suit, (ver. 171.42.74a)[6] with the data cut-off up to 1 Å. The data reduction procedure was similar to one described in section 8.4.3, however unit cell indexation required introducing initial unit cell from X-ray data. The crystal structure determination was eventually performed for dataset collected for unmerged dataset from one best crystal, since its completeness was close to 100%. Attempts to use XDS software were unsuccessful due to failed unit cell indexing.

For this crystal the total number of electrons per molecule was calculated. The number of molecules was taken from crystal size (3 x 1 x 1 μm) and its density from X-ray structure (1.205 g/cm^3).

$$N = N_A \frac{d \cdot V}{M} = 6.02 \cdot 10^{23} \frac{1.205 \, {}^{g}/_{cm^3} \cdot 3\mu m^3}{490.84 \, {}^{g}/_{mol}} = 4.43 \cdot 10^9$$

The illuminated area was assumed to be equal during the whole tilt series and calculated as a surface of the biggest crystal face, i.e. 3 μm^2 and the total irradiation time was equal to 50 seconds (1tilt series) what eventually led to approximately 100 molecules per electron according to the following equation:

$$\frac{N}{N_{e-}} = \frac{N}{D \cdot A \cdot t} = \frac{N}{0.003\, {e^-}/{\text{Å}^2 s} \cdot 3\mu m^2 \cdot 50\, s} = \frac{4.43 \cdot 10^9}{4.50 \cdot 10^7} \approx 100$$

Comparing of this value to the ones obtained in the grid irradiation series suggest that the microcrystal underwent 100% conversion, since according to the previous calculations 1 electron can cause up to ~90,000 reactions. However it has to be taken into account that the thick compound pellet is opaque to electrons, while microcrystal is transparent for most of them. The real number of electrons interacting with the sample is thus unknown, however much lower. For example assuming 99.9% transmission, which is not unlikely, the total number of molecules per electron would be 1000 times higher, i.e. 100,000.

Attempts to determine MicroED crystal structure of Dewar Benzene 1. The structure does not solve using all the most common approaches including direct methods, intrinsic phasing, dual space and charge flipping, however the maxima on the normalized structure factor map correspond very loosely to the atomic positions in structure determined from X-ray. In order to obtain approximate model of the crystal structure the isomorphous replacement method was used. The initial atomic positions were taken from X-ray structure and the adamantyls, dewar benzenes and COO groups were constrained as independent rigid bodies and all the hydrogen atoms were removed. After first refinement cycles the minor components in disordered dewar benzenes were removed due to their unstable behavior. In the next step the weighting scheme and extinction corrections were applied and afterwards the rigid bodies constraints were removed in order to refine the atomic positions freely. In the last refinement cycle the hydrogen atoms were constrained in corresponding positions. The refinement was performed using more forgiving Levenberg-Marquardt method,[7] because least-squares refinement on F^2 always led to negative definition of atomic displacement parameters (ADP). Even though some of the bond lengths, valence angles and ADP's differ significantly from physical values no more interventions were applied to the model to prove the stability of the refinement and exclude the possibility of overfitting, however

it is possible that the stability of the model is due to landing in the false minimum during the Levenberg-Marquardt refinement.

Nevertheless, the final model has good refinement statistics for MicroED data (e.g. R1=23.45%, GooF=0.939) and it resembles the X-ray structure, Fig. S9, however how close to the truth, unfortunately it remains ambiguous, e.g. the overall data quality and low resolution do not allow to establish reliably either the crystal contain Dewar benzene or Hückel benzene or mixture of both. Nonetheless, it proves that the collected low dose MicroED data contain meaningful information which are simply impossible to extract without X-ray structure what leads to more general conclusion that the common problems with microED crystal structure determinations might be associated with chemical events rather (Dewar benzene **1** to benzene **2** transformation in this particular case) than classical crystal damage due to inelastic events, more frequently than it seems.

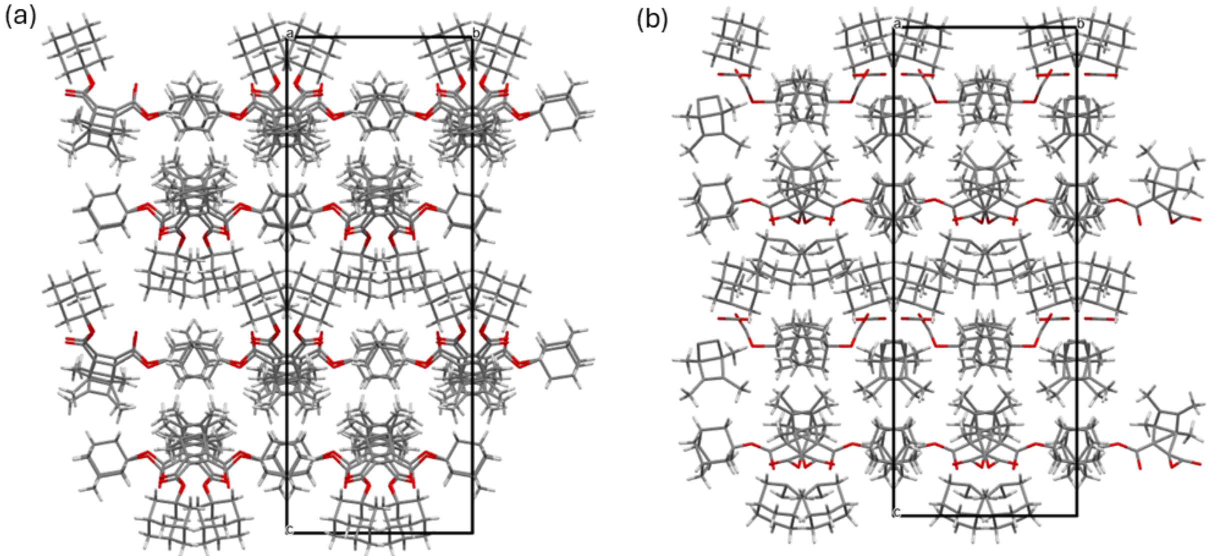

Figure S9. (a) X-ray structure and (b) Micro-ED model of Dewar benzene **1** at 100K

8.3.3 MicroED crystal structure of recrystallized Benzene 2

Despite many tries we were not able to grow crystals of benzene **2** big enough for conventional X-ray structure analysis hence the use of micro-ED structure analysis for benzene **2** crystals was not dictated by the nature of this publication but by the actual necessity. The final ED structure was deposited within CCDC with #2352506.[5]

Data collection at RT. Even though the benzene **2** is unambiguously much more resilient to the electron damage then Dewar benzene **1**, it is still losing crystallinity relatively fast and allows to collect only ca. 20 frames containing meaningful data from a single crystal, Fig. S10., and the tries of crystal structure determination at RT were unsuccessful.

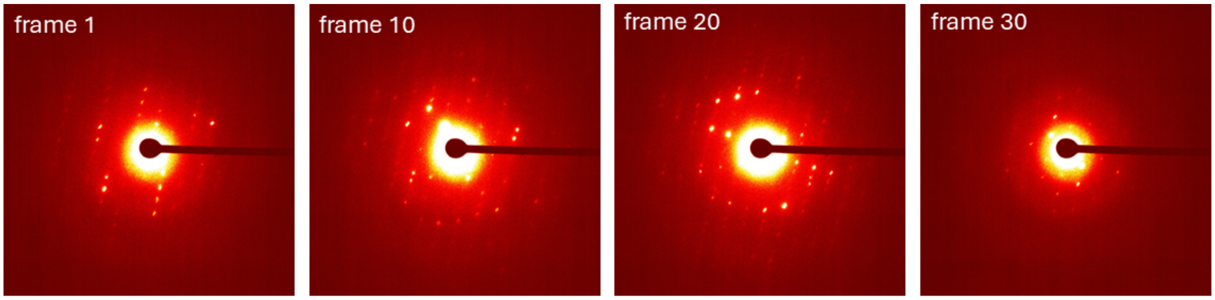

Figure S10. Diffraction images for single tilt series of Benzene **2** crystal at RT.

Data collection and reduction at 100K. Samples of benzene **2** were recrystallized from hexane to form small needle shaped crystals organized into bundles stuck together along the longest crystal faces on the sides of the vessel. The sides of the vessel were scratched with metal spatula to free small crystalline chips of the compound and the TEM grid was deposited inside the vessel which was vigorously shaken for few minutes. This ensured satisfactory micro/nanocrystal distribution on the grid (see Fig. S11).

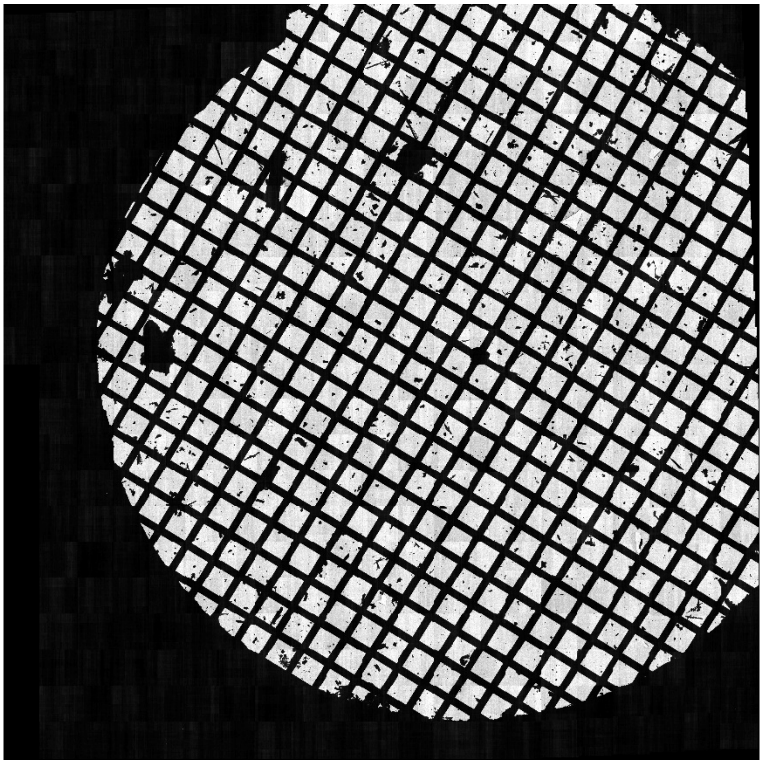

Figure S11. The TEM grid contains benzene **2** crystals prepared for Micro-ED measurements.

The grid was placed on the Cryo-transfer holder and cooled down to 100K after the transfer to the microscope. The screening was performed based on the TEM image in Fig. S11 but the exact position of the beam relative to the crystal was determined in diffraction mode. The single-tilt data collection was performed for ca. 50 crystals, usually within 110 degrees range, however for some of them the range was limited by overshadowing elements (copper, big clusters of crystals). The raw diffraction images were transferred to the CrysAlisPro software suit (ver. 171.42.74a)[6] where initial visual evaluation and preliminary unit cell indexation of electron diffraction images was performed based on which 10 best datasets was picked for data reduction. For each dataset the instrument model was refined iteratively, including refinement of the rotation axis, equivalent of the kappa angle in 4-circle goniometer and sample-detector distance. The data reduction with the cut-off at 0.81Å for each dataset was executed 24 times for various integration parameters combinations (mask size, background evaluation etc). This narrowed the datasets to 5 most promising, based on Rint and I/σ(I), which were merged together and rescaled. Different merging combinations were solved and crystal structures were determined and compared (e.g. crystals 1+2+3+4+5, 1+2+3+4, 2+3+4, 1+3+4, 2+3 etc.) We found that merging 3 or less datasets didn't allow high completeness (at best around 85%) and there is no significant difference between

merging 4 or 5 datasets, thus we decided to choose the structure based on more redundant data (i.e. from 5 crystals rather than 4). After first merging the scale factor in the function of frame number was re-evaluated and additional frames with strongly deviating scale factor were removed while the data completeness was maintained. The exemplary TEM images and ED patterns for all five crystals used for structure determination are shown in Fig. S12.

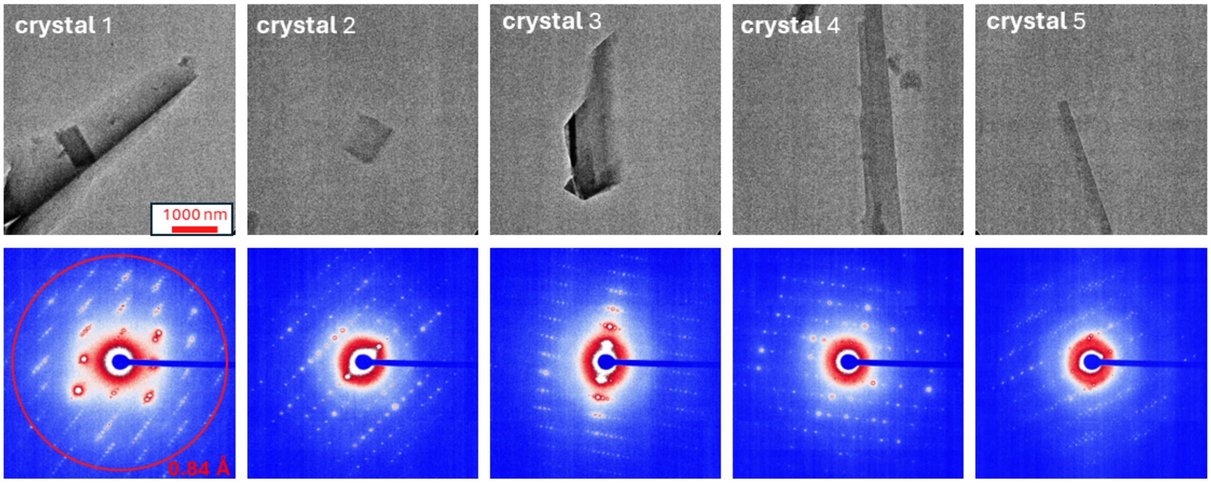

Figure S12. TEM crystal images and arbitrary chosen Electron diffraction pattern for crystals used for benzene **2** structure determination at 100 K.

Space group determination

The Wilson and cumulative intensities plots both suggested non-centrosymmetric space group and the structure can be solved both in Pc and $P2_1$ space group with low R_1 (~14-15%), however the bond lengths for these structures deviates strongly from the expected and systematic absences both for 2_1 screw axis and c glide plane are present in diffraction images. The R_{int} for $P2_1$, Pc and $P2_1/c$ space groups remains the same - lower symmetry does not improve it. For these reasons the $P2_1/c$ space group was found to be the correct one.

Datasets merging. It is possible to solve the structure from a single dataset for 4 out of 5 crystals using direct methods in ShelXS, for crystal 1 it was impossible due to low tilt range. These single tilt structures are characterized by lower R_{int} and completeness, and goodness of fit strongly deviating from 1 comparing to the merged dataset. Due to merging datasets for crystals of different sizes and quality in the final dataset the R_{int} value significantly increased, however the final R_1, goodness of fit, ADP's, completeness and overall quality of the structure including bond lengths and valence angles is the best for merged dataset.

Table S3. Micro-ED crystal structures data

Type of measurement	μ-ED, 300 keV					
Structure	Crystal 1	Crystal 2	Crystal 3	Crystal 4	Crystal 5	Merged (final)
Frames #	64	65	99	92	98	418
a [Å]	6.56(14)	6.61(17)	6.47(1)	6.43(13)	6.45(14)	6.44(13)
b [Å]	18.8(4)	19.19(19)	18.99(11)	18.7(2)	18.9(3)	18.8(2)
c [Å]	20.49(14)	20.8(3)	20.14(17)	20.37(18)	20.2(2)	20.38(19)
β [°]	92.21(11)	92.91(19)	92.72(11)	92.38(12)	92.99(17)	92.49(13)
Volume [$Å^3$]	2524(7)	2630(81)	2473(47)	2452(59)	2457(68)	2459(60)
Reflections collected	5887	6697	9527	9591	8291	39809
Reflections independent	2479	3086	3010	3468	3071	4862
Reflections observed	1830	2187	2506	2729	1752	4438
Completeness [%]	51.6	61.4	62.4	72.4	63.8	99.3
R_{int}	0.1022	0.0878	0.1186	0.1306	0.1487	22.66
R [$F^2>2\sigma(F^2)$], wR, S	0.2276,0.5325, 1.976	0.1863,0.4851, 1.677	0.2234,0.5122, 2.006	0.2280,0.5350, 1.989	0.1651,0.4453, 1.314	0.1931,04032, 1.196
$\Delta\rho_{max}$, $\Delta\rho_{min}$	0.66, -0.71	0.69, -0.73	0.53, -0.62	0.68, -0.76	0.84, -0.79	0.17, -0.22

*For all crystals: crystal system: monoclinic, space group: $P2_1/c$, temperature=100K, Z,Z'=4,1

The final structure was solved using kinematical approximation by direct methods in ShelXS[3] and refined in ShelXL by the full-matrix least-squares on F^2 approach[4] using electron atomic scattering factors[8] in Olex2. [7]

All non-hydrogen atoms were refined anisotropically. The restraints on sphericity of ADP's of atoms C2, C12, C13, C20 and C27 and on equality of ADP's of carbon atoms in phenyl ring (C1→C6) were applied. The hydrogen atoms were fixed geometrically in their corresponding positions using distances from neutron diffraction data (1.099, 1.092 and 1.077 Å for CH, CH2 and CH3, respectively). Additionally, 18 of the most disagreeable reflections were omitted from the refinement (final data/parameter ratio 13.5). The extinction correction was applied with the high final value of 795 which is not unusual in kinematical approximation from data obtained from

electron diffraction. The asymmetric unit with labeling scheme and the unit cell content are shown in Fig. S13.

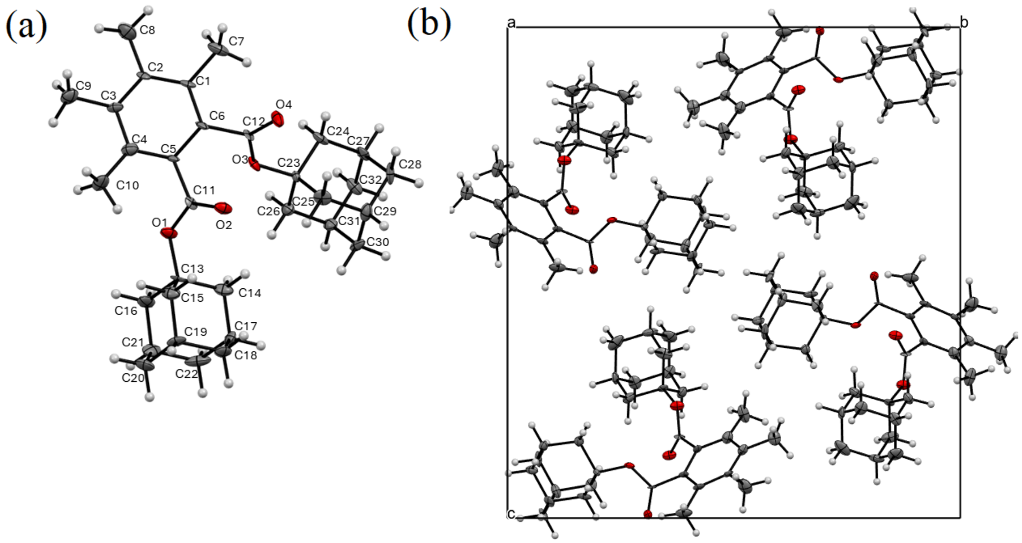

Figure S13. (a) Molecular structure with labeling scheme and (b) unit cell content along [100].

8.3.4 Structures comparison

With certainty the crystal structure of recrystallized benzene **2** does not corresponds to the structure of benzene **2** obtained in-situ by irradiation of crystalline Dewar benzene **1**, since recrystallization allows the optimal conformation of the benzene **2** molecules what is not the case for in-situ obtained product, restrained by tight crystal packing. Nevertheless, comparison of both benzene **2** and Dewar benzene **1** structures sheds light on the nature of crystal damage induced by the reaction and as it can be seen the molecular structures differ significantly from one another, not only in the reaction center, Fig. S14a. This is due to huge structural transformation during the reaction associated with the change from V-shaped, more globular geometry of Dewar benzene **1** to flattened geometry of aromatic benzene **2**. This is schematically visualized in Fig. 14b. This kind of transformation would require either exceptional elastic properties or big reaction cavity of crystals to remain high crystallinity, and since none of these apply the reaction causes sever crystal damage.

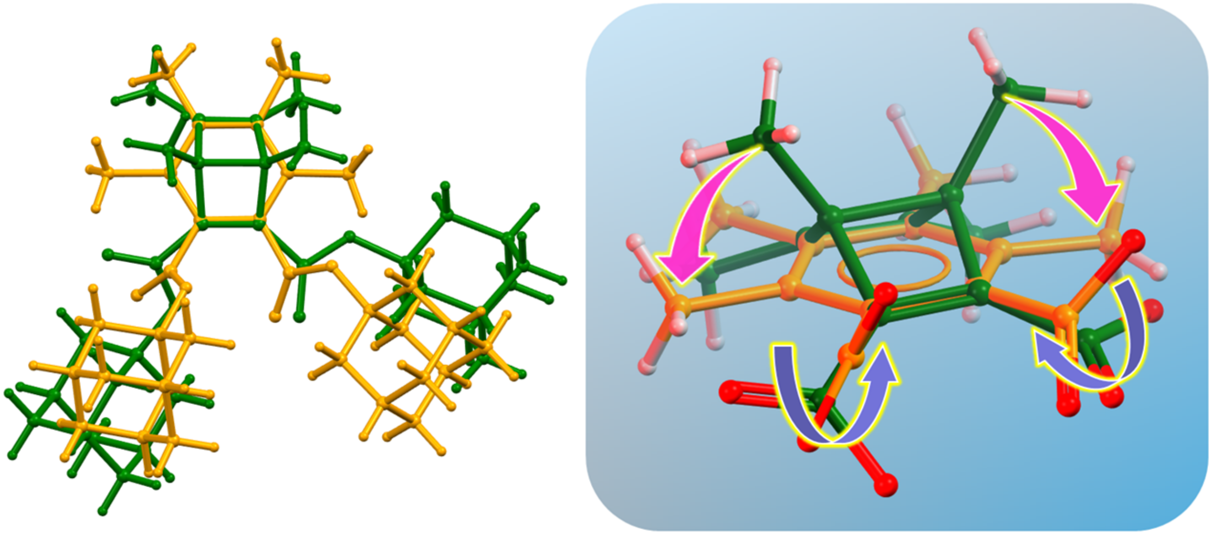

Figure S14. (a) Overlap of Dewar benzene **1** and benzene **2** molecules taken from crystal structures and (b) schematic representation of transformation from Dewar benzene **1** (green) to benzene **2** (orange). Cleavage of the central bond in Dewar benzene **1** leads to arc shifts of methyl groups marked with pink arrows, what in turn, due to the steric repulsion forces the rotation of carboxylic groups, marked with blue arrows.

9. **NMR**:

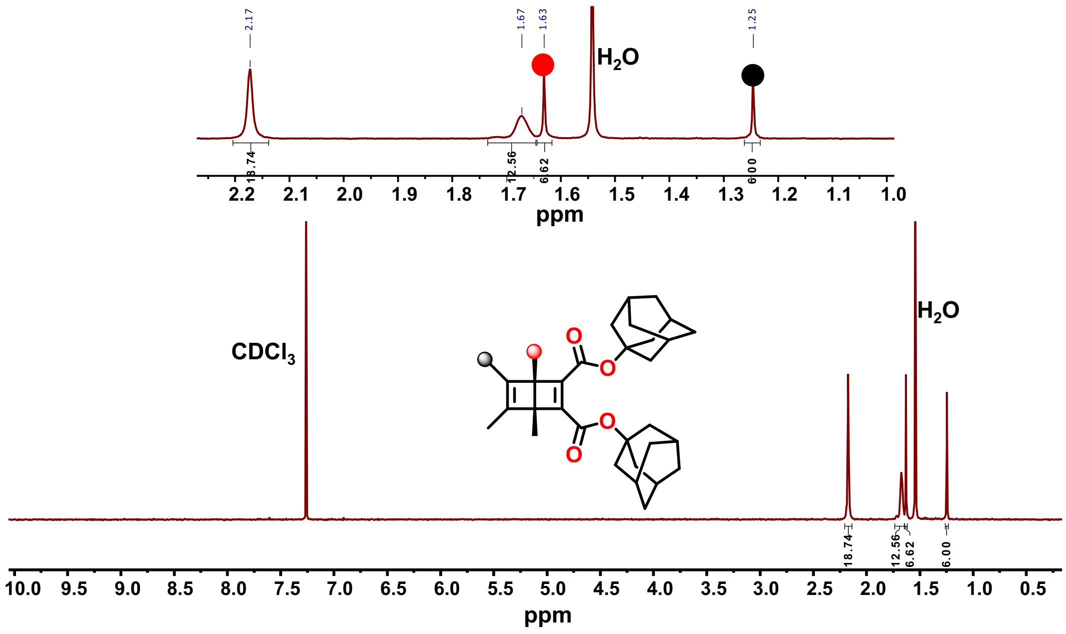

Figure S15. ^{1}H NMR spectrum of Dewar benzene **1** in $CDCl_3$ (400 MHz, 298 K).

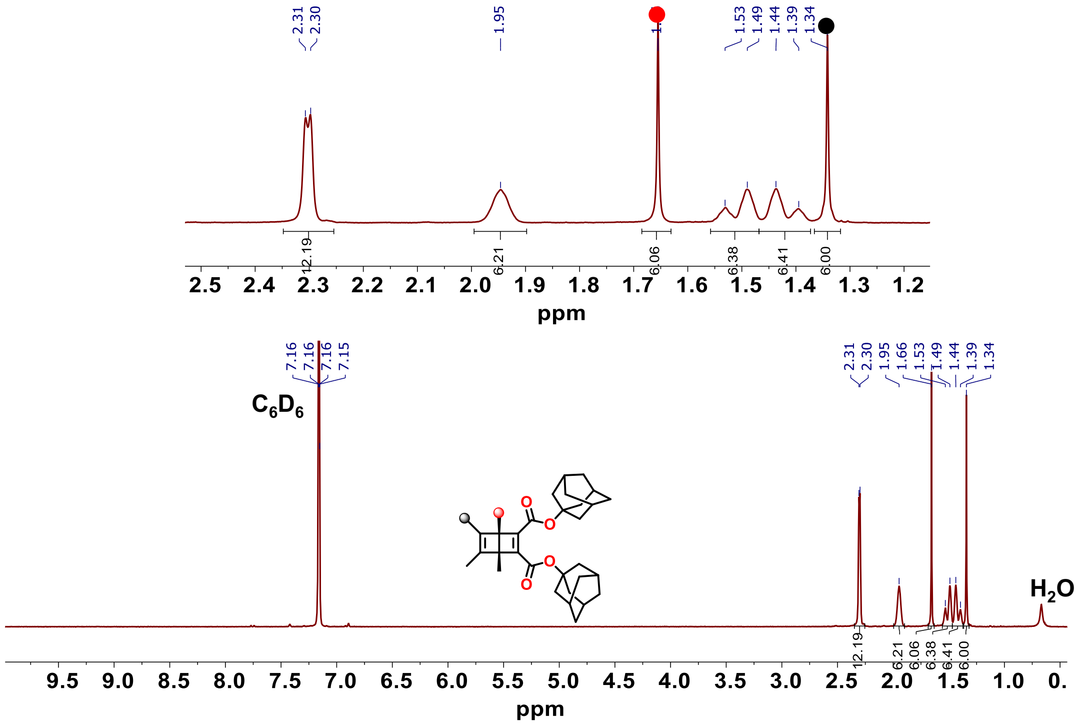

Figure S16. ^{1}H NMR spectrum of Dewar benzene **1** in C_6D_6 (400 MHz, 298 K).

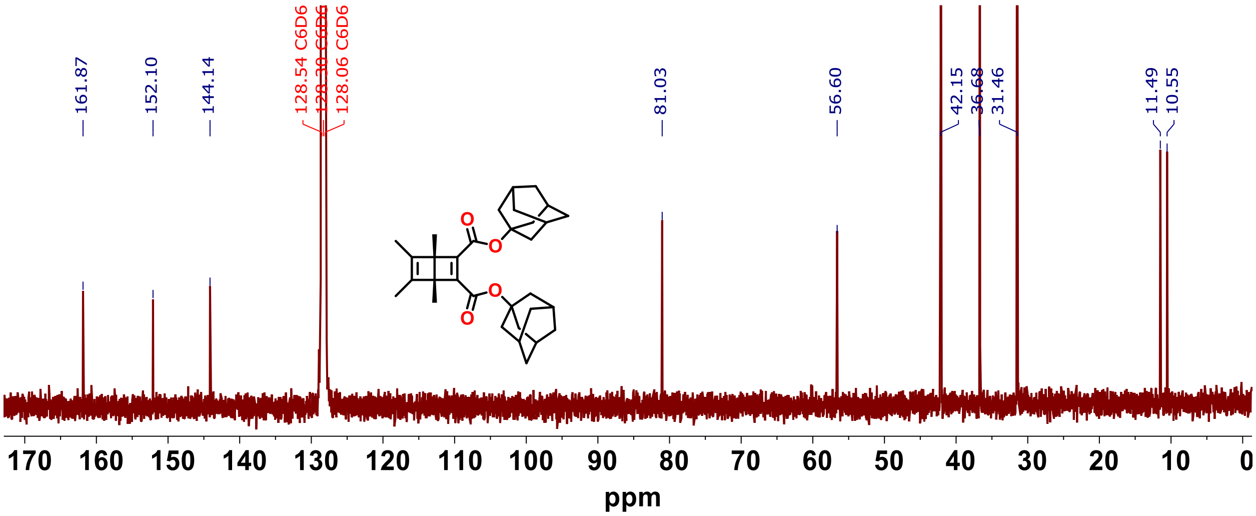

Figure S17. ^{13}C NMR spectrum of Dewar benzene **1** in C_6D_6 (400 MHz, 298 K).

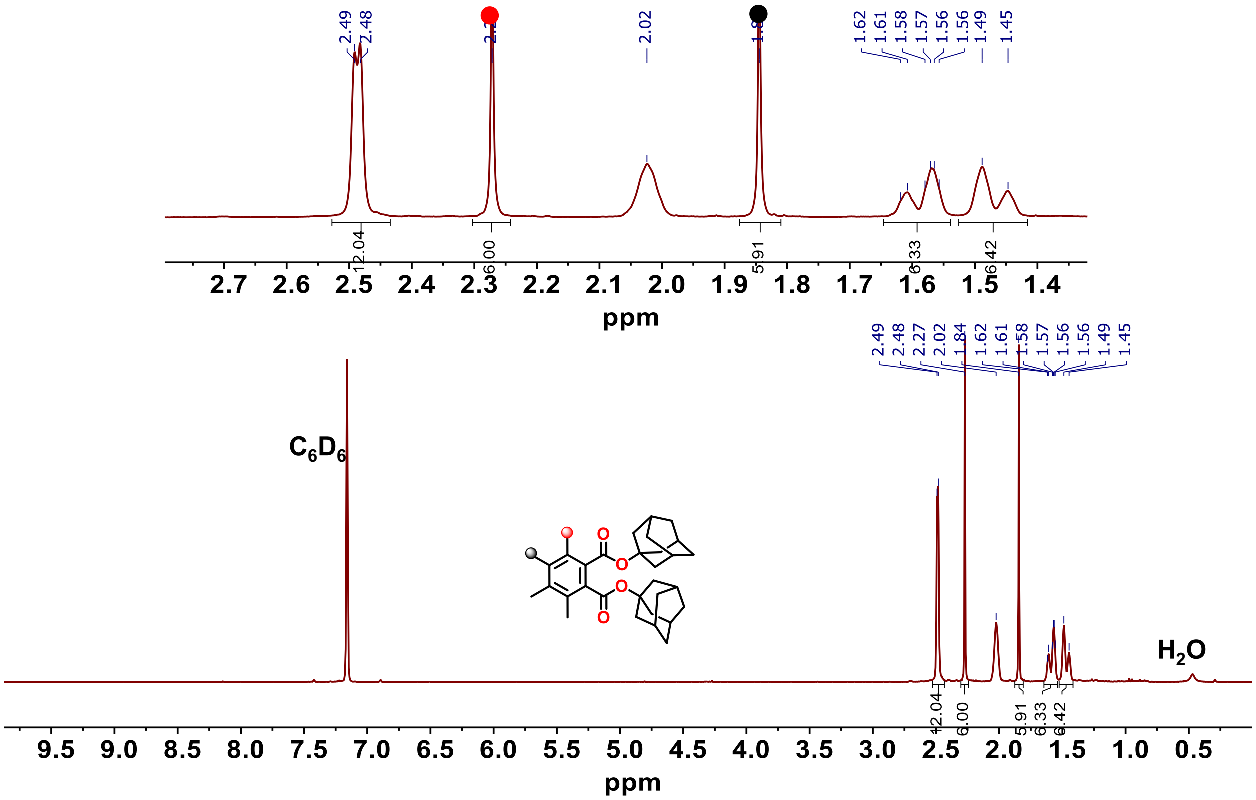

Figure S18. ^{1}H NMR spectrum of benzene **2** in C_6D_6 (400 MHz, 298 K).

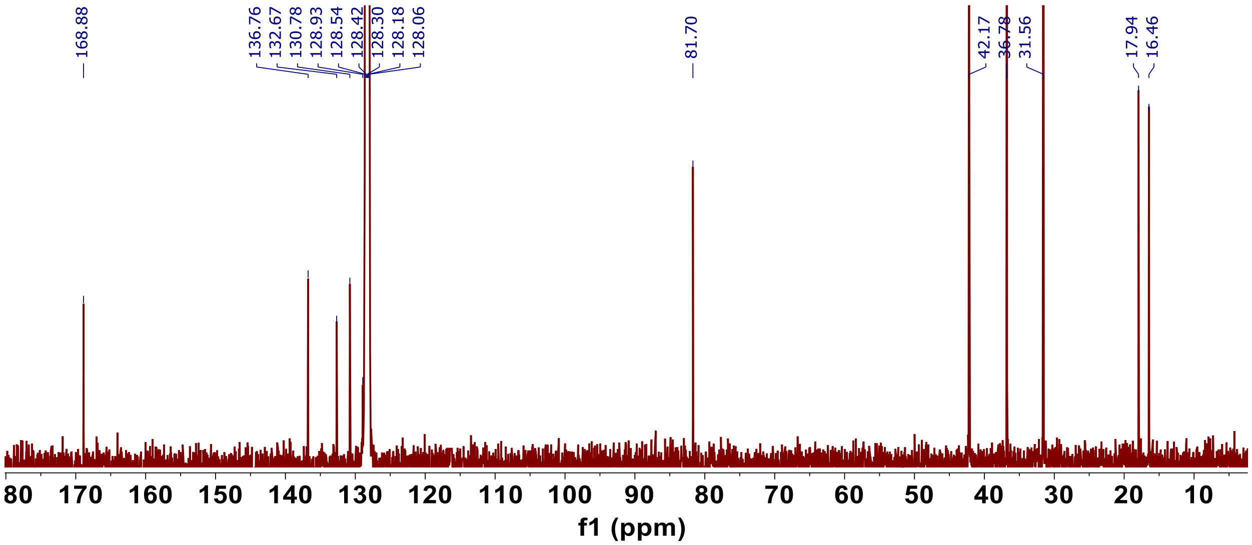

Figure S19. ^{13}C NMR spectrum of benzene **2** in C_6D_6 (400 MHz, 298 K).

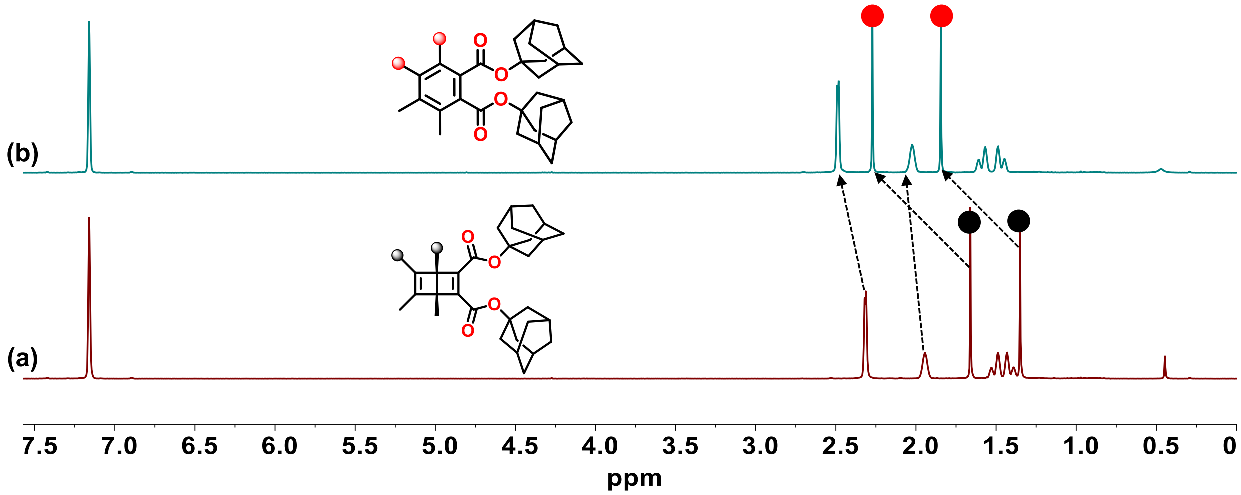

Figure S20. Comparison ^{1}H NMR spectra (400 MHz, 298 K) of (a) Dewar benzene **1** (b) benzene **2** in C_6D_6

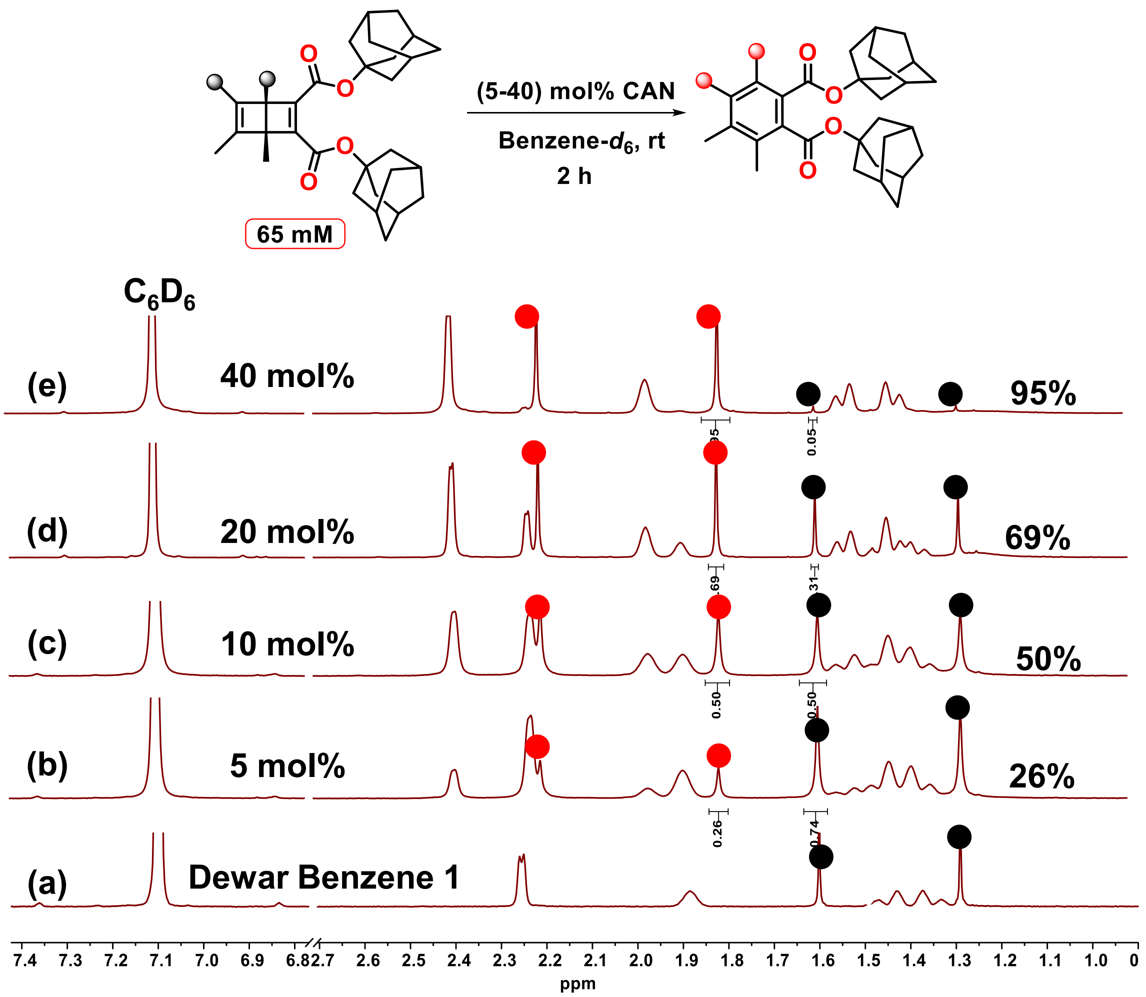

Figure S21. ^{1}H NMR spectra (400 MHz, C_6D_6, 298 K) of (a) Dewar Benzene **1** (a) reaction of Dewar benzene **1** with 5 mol% ceric ammonium nitrate (CAN) at 25 °C for 2 h in C_6D_6 (c) reaction of Dewar benzene **1** with 10 mol% CAN at 25 °C for 2 h in C_6D_6. (d) reaction of Dewar benzene **1** with 20 mol% CAN at 25 °C for 2 h in C_6D_6. (e) reaction of Dewar benzene **1** with 40 mol% CAN at 25 °C for 2 h in C_6D_6. The black circles correspond to Dewar benzene **1**, whereas the red circles represent product benzene **2**.

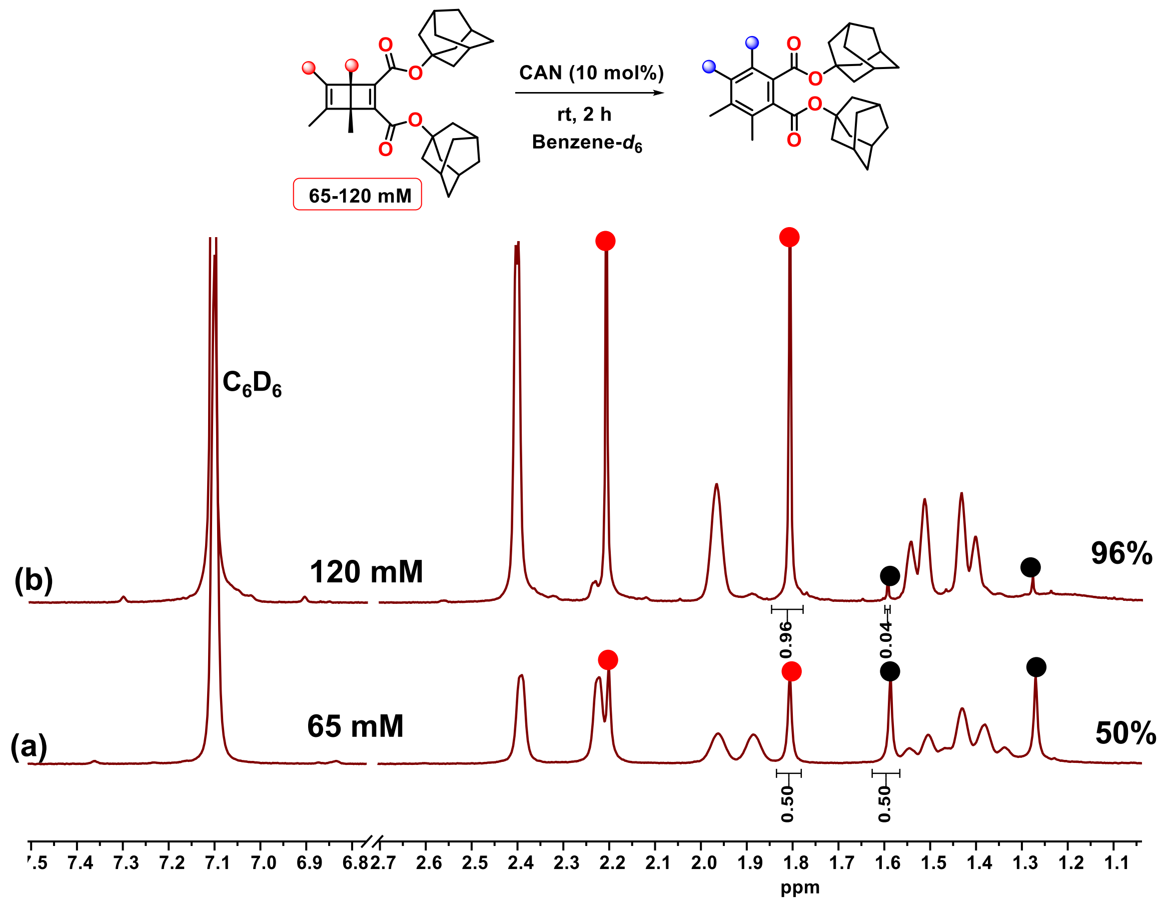

Figure S22. ^{1}H NMR spectra (400 MHz, C_6D_6, 298 K) of reaction with ceric ammonium nitrate (CAN) (10 mol%) at 25 °C for 2 h in C_6D_6 (a) 65mM Dewar benzene **1** (b) 120 mM Dewar benzene **1**. The black circles correspond to Dewar benzene **1**, whereas the red circles represent product benzene **2**.

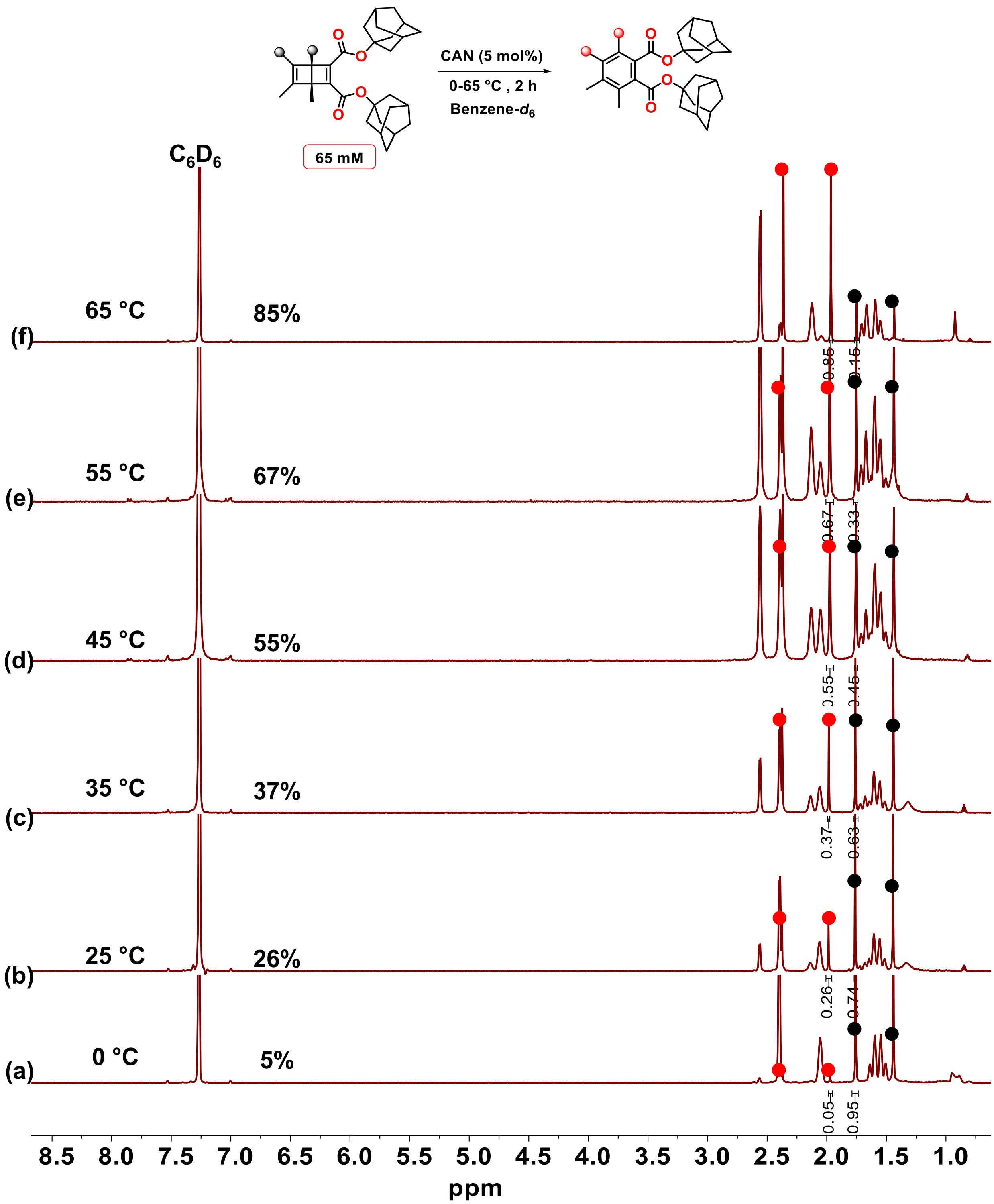

Figure S23. ^1H NMR spectra (400 MHz, C_6D_6) of a reaction with Dewar benzene **1** (65 mM) and ceric ammonium nitrate (CAN) (5 mol%) for 2 h in C_6D_6 at temperature (a) 0 °C (b) 25 °C (c) 35 °C (d) 45 °C (e) 55 °C (f) 65 °C. The black circles correspond to Dewar benzene **1**, whereas the red circles represent product benzene **2**.

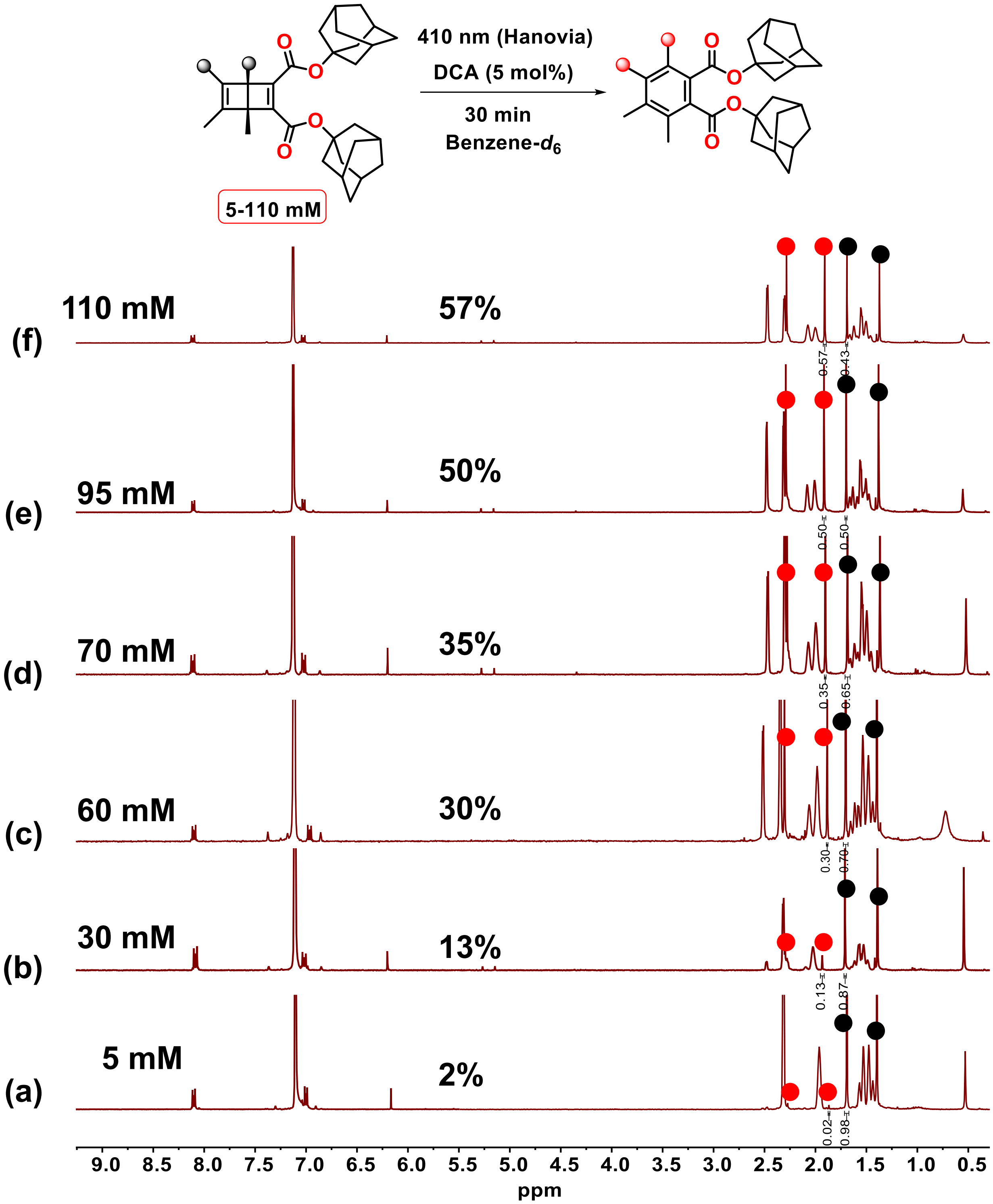

Figure S24. ^{1}H NMR spectra (400 MHz, C_6D_6) of a reaction with 9,10-dicyanoanthracene (0.25 mmol) and Dewar benzene **1** (a) 5 mM (b) 30 mM (c) 60 mM (d) 70 mM (e) 95 mM (f) 110 mM at 25 °C for 2 h with 410 nm filter using Hanovia (Hg lamp). The black circles correspond to Dewar benzene **1**, whereas the red circles represent product benzene **2**.

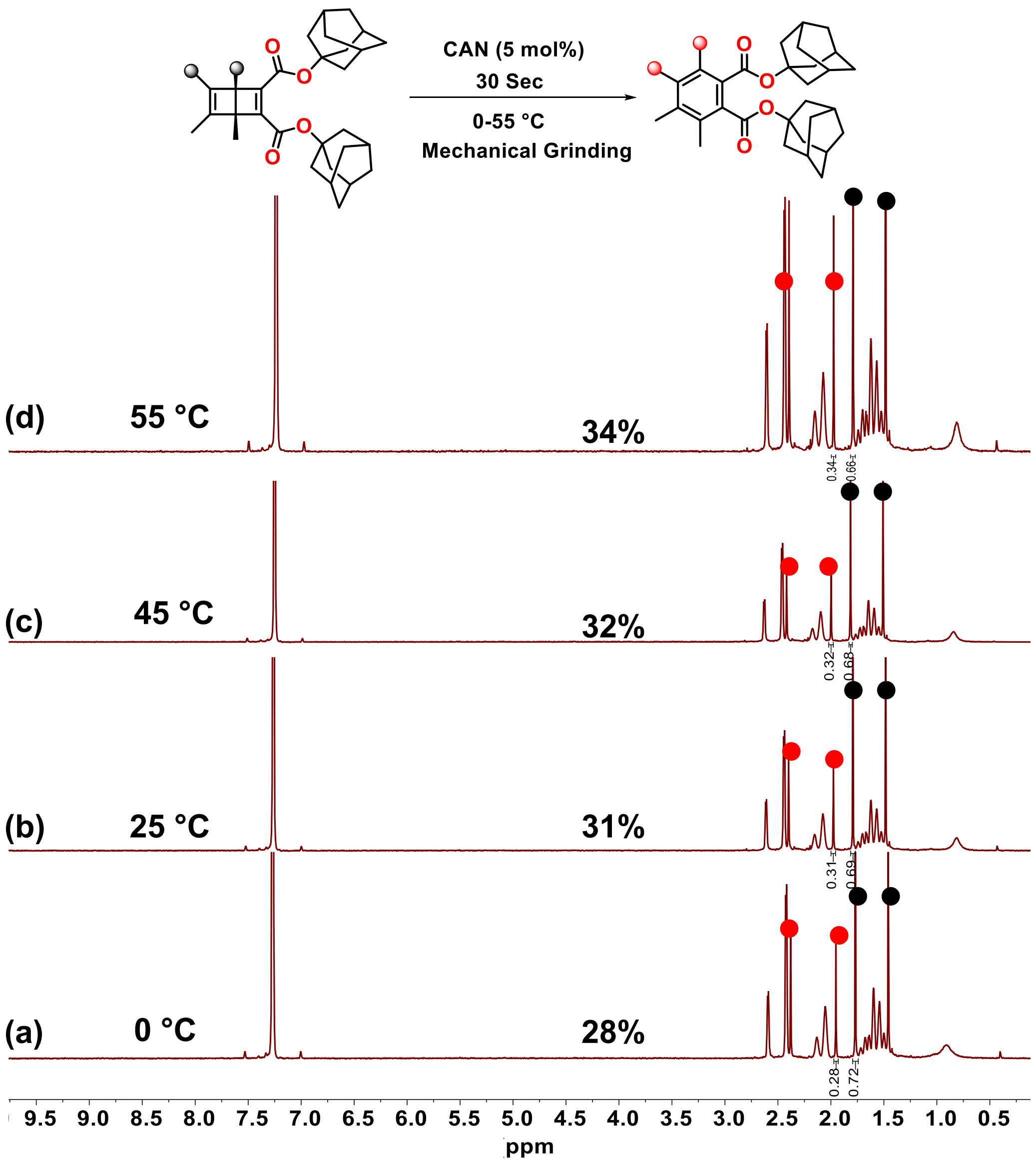

Figure S25. ^{1}H NMR spectra (400 MHz, C_6D_6) of a mechano-chemical reaction of Dewar benzene **1** (10 mg) and ceric ammonium nitrate (CAN) (5 mol%) for 30 sec at temperature (a) 0 °C (b) 25 °C (c) 45 °C (e) 55 °C. The black circles correspond to Dewar benzene **1**, whereas the red circles represent product benzene **2**.

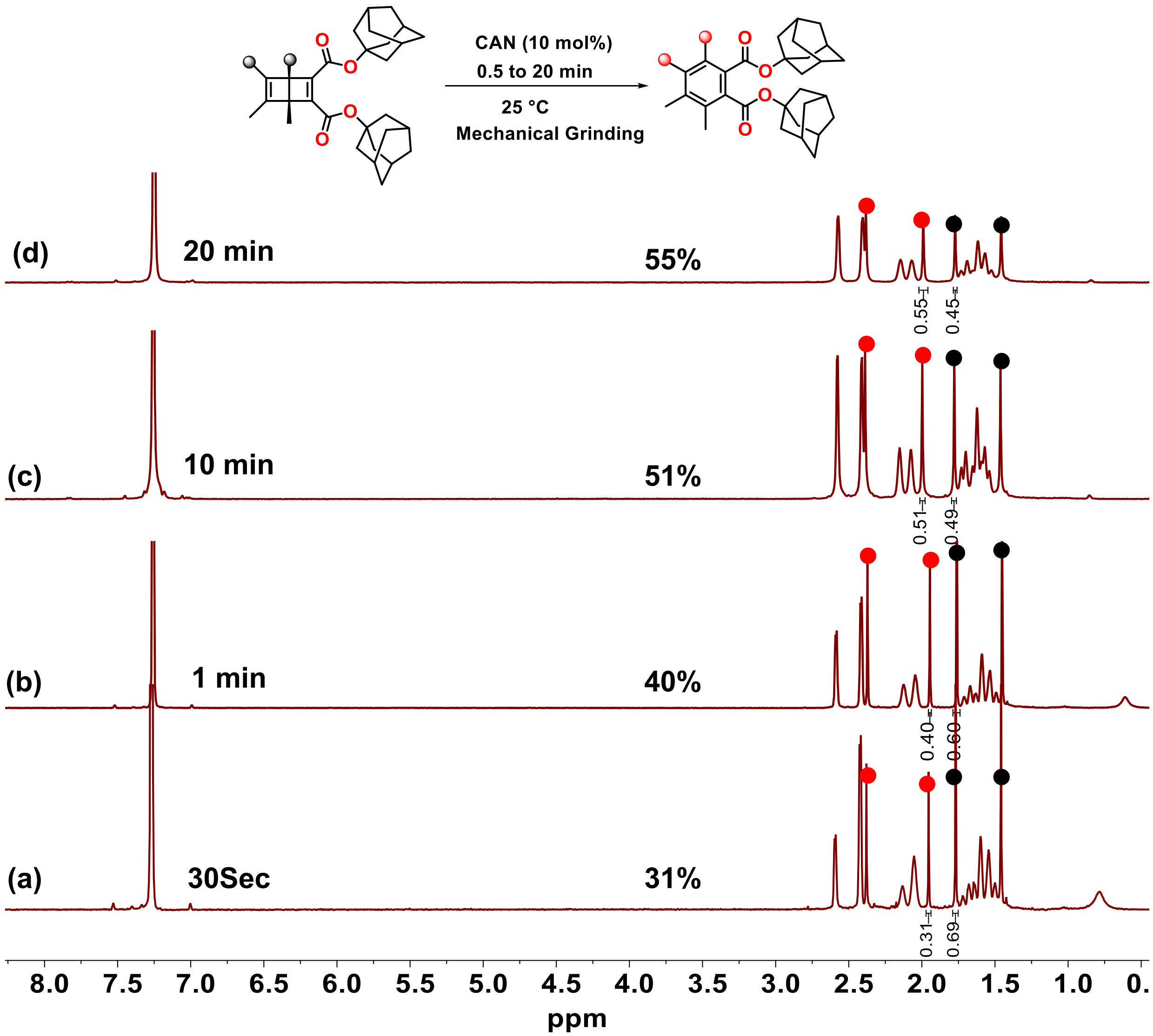

Figure S26. ^{1}H NMR spectra (400 MHz, C_6D_6) of a mechano-chemical reaction of Dewar benzene **1** (10 mg) and ceric ammonium nitrate (CAN) (10 mol%) at 25 °C for (a) 30 sec (b) 1 min (c) 10 min (20 min). The black circles correspond to Dewar benzene **1**, whereas the red circles represent product benzene **2**.

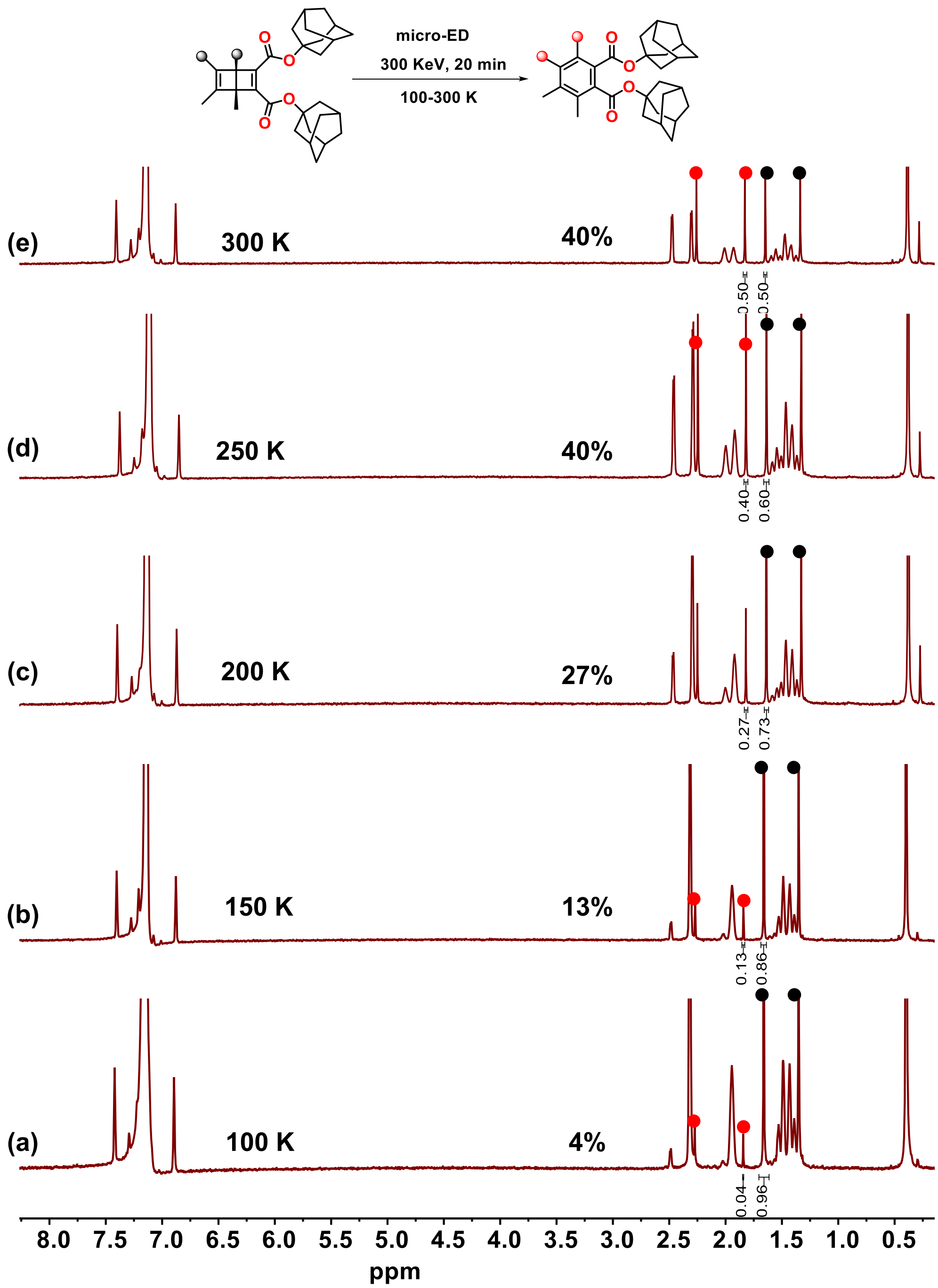

Figure S27. ^{1}H NMR spectra (400 MHz, C_6D_6) of micro electron diffraction beam induced isomerization of Dewar benzene **1** (~0.2 mg) to benzene **2** at (a) 100 K (b) 150 K (c) 200 K (d) 250 K (e) 300 K using 300 keV beam energy with dose of 6.00×10^{-5} for 20 minutes exposure time. The black circles correspond to Dewar benzene **1**, whereas the red circles represent product benzene **2**.

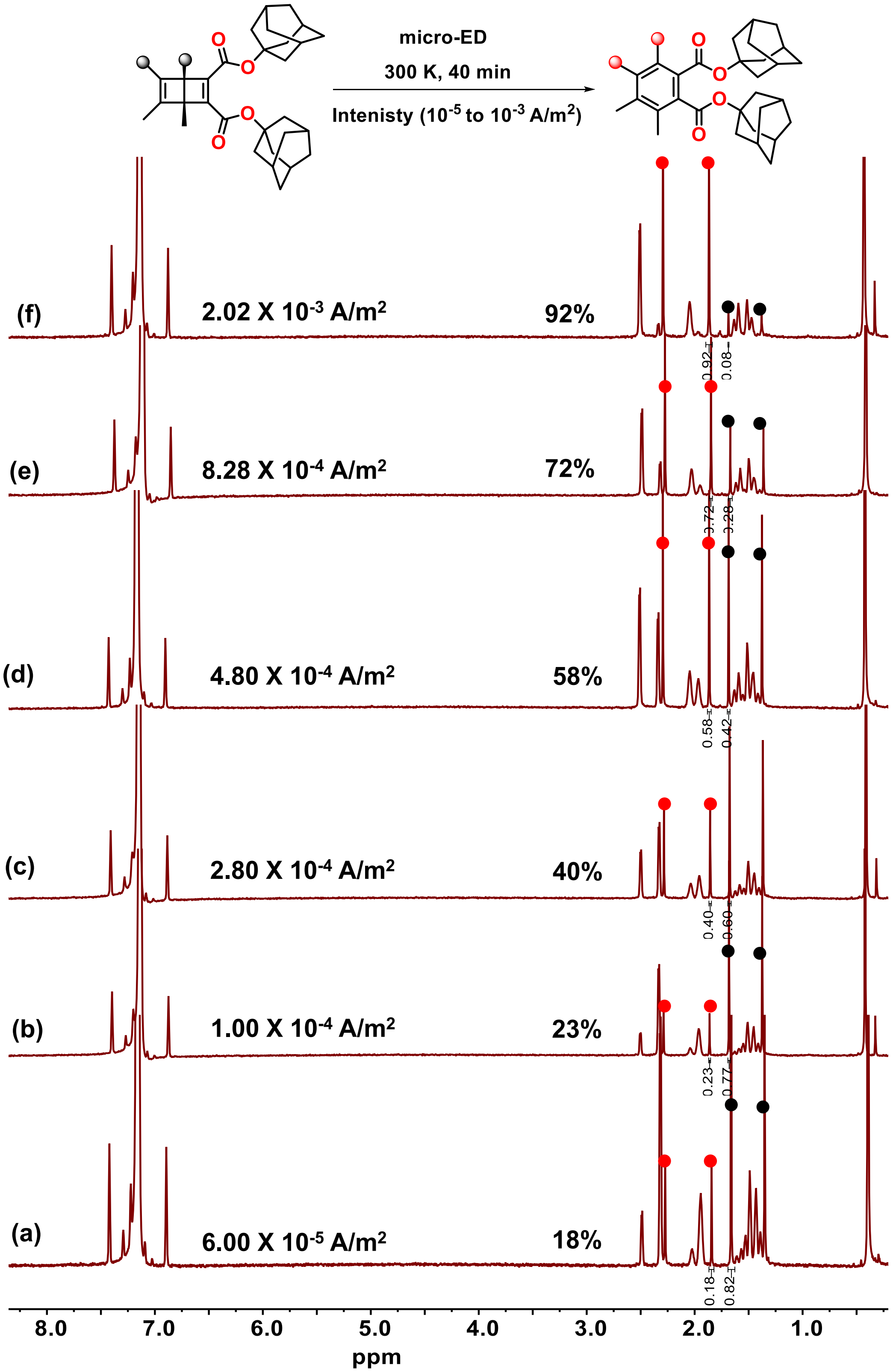

Figure S28. ^{1}H NMR spectra (400 MHz, C_6D_6) of micro electron diffraction beam induced isomerization of Dewar benzene **1** (~0.2 mg) to benzene **2** at 300 K using 300 keV electron beam energy with dose rate of (a) 6.00×10^{-5} Am^{-2} (b) 1.00×10^{-4} Am^{-2} (c) 2.80 × 10^{-4} (d) 4.80×10^{-4} (e) 8.28×10^{-4} Am^{-2} (f) 2.02×10^{-3} Am^{-2} for 20 minutes exposure time. The black circles correspond to Dewar benzene **1**, whereas the red circles represent product benzene **2**.

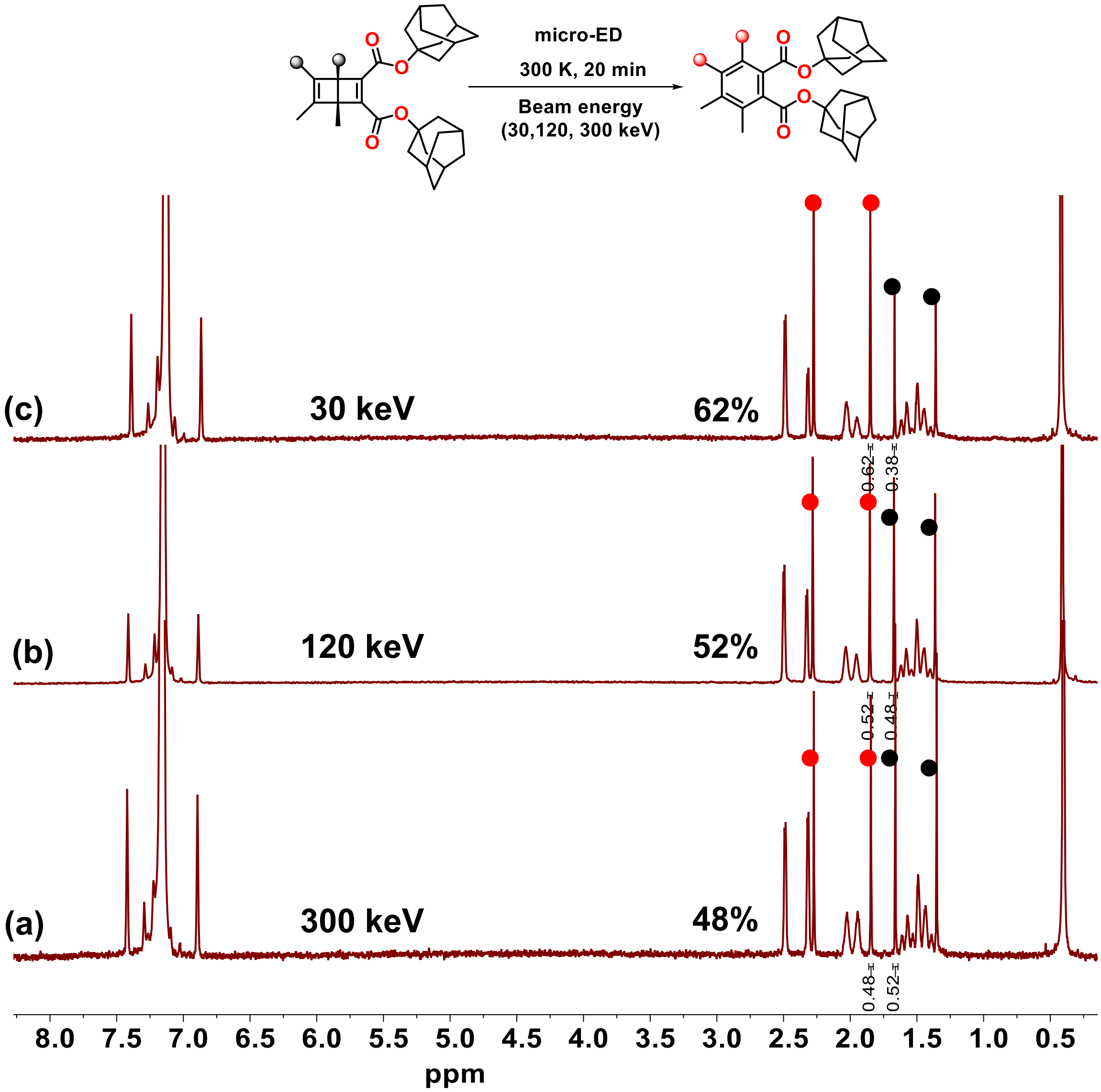

Figure S29. ^{1}H NMR spectra (400 MHz, C_6D_6) of micro electron diffraction beam induced isomerization of Dewar benzene **1** (~0.2 mg) to benzene **2** at 300 K using electron beam energy (a) 300 keV (b) 120 keV (c) 30 keV for 20 minutes exposure time. The black circles correspond to Dewar benzene **1**, whereas the red circles represent product benzene **2**.

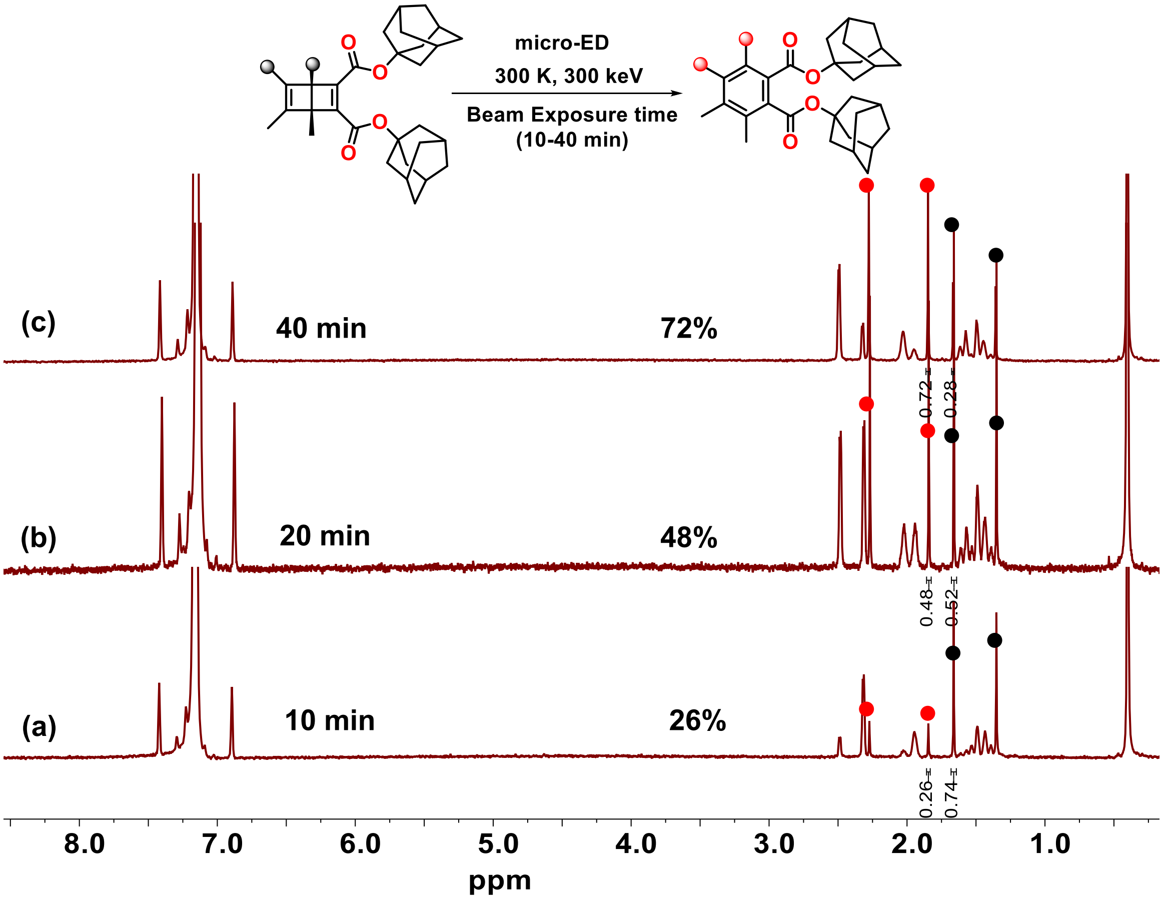

Figure S16. ^{1}H NMR spectra (400 MHz, C_6D_6) of micro electron diffraction beam induced isomerization of Dewar benzene **1** (~0.2 mg) to benzene **2** at 300 K using 300 keV electron beam energy with dose rate of 5.95×10-5 A/m^2 for (a) 10 minutes (b) 20 minutes (c) 40 minutes exposure time. The black circles correspond to Dewar benzene **1**, whereas the red circles represent product benzene **2**.

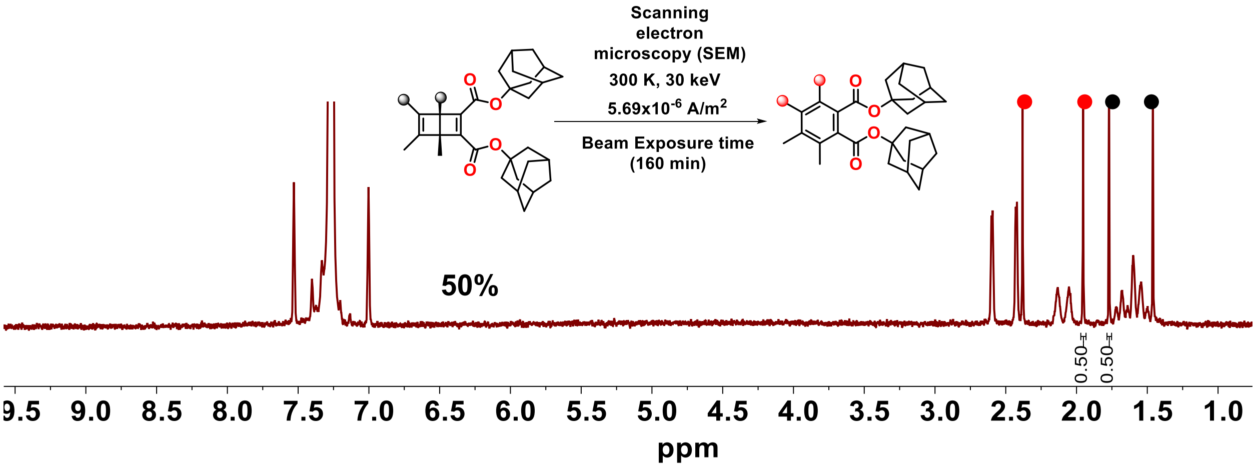

Figure S30. ^{1}H NMR spectra (400 MHz, C_6D_6) of scanning electron microscopic isomerization of Dewar benzene **1** (~0.2 mg) to benzene **2** at 300 K using 30 keV electron beam energy with dose rate of 5.69×10-6 A/m^2 for 2 h 40 min. The black circles correspond to Dewar benzene **1**, whereas the red circles represent product benzene **2**.

10. Electrochemistry

10.1 Electrochemical methods: Electrochemistry was performed using either a Gamry Instruments Interface 1000-E or a CH Instruments 760E potentiostat. A glassy carbon electrode was utilized as the working electrode, and platinum for the counter and pseudo reference. Potentials were calibrated using a ferrocene/ferrocenium (Fc/Fc^+) internal standard. All electrodes were purchased from CH Instruments. The glassy carbon electrode was cleaned via polishing with diamond paste, and both the glassy carbon working and platinum counter/pseudo reference electrodes were cleaned via sonication. The electrolyte solution consisted of 0.1 M tetrabutylammonium hexafluorophosphate (TCI Chemicals, > 98.0%, used without further purification) in acetonitrile. Both Dewar benzene **1** and benzene **2** was prepared as 2 mM for all electrochemical process.

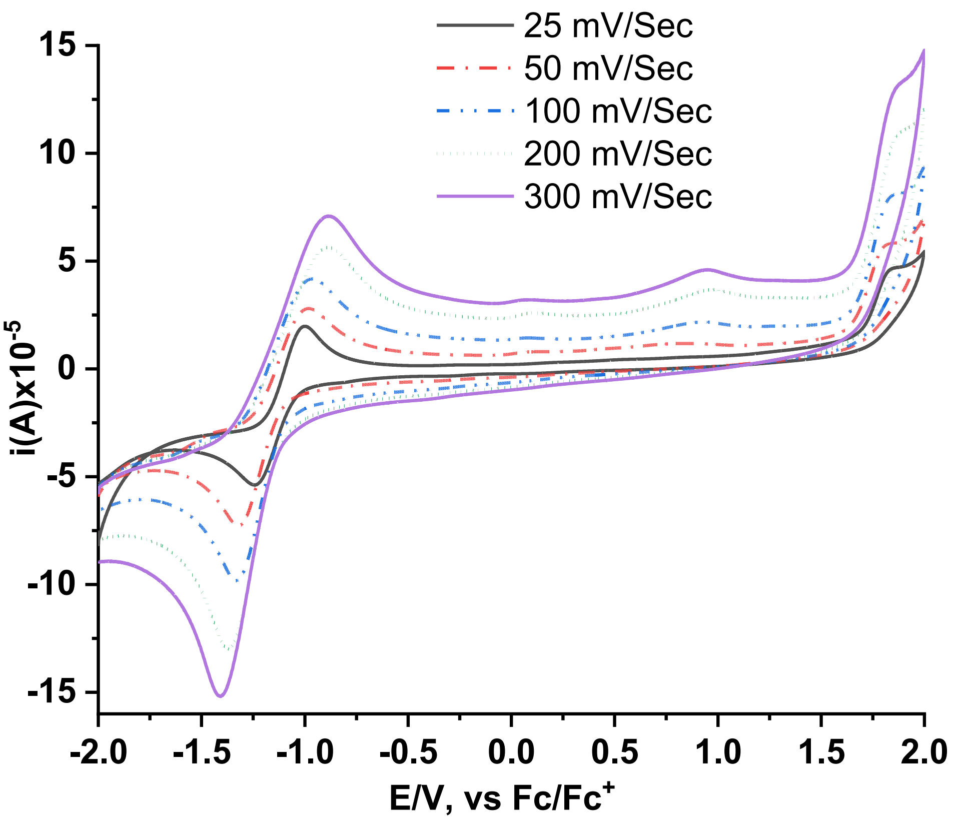

Figure S31. Cyclic voltammograms Dewar benzene **1** on glassy carbon electrodes in ambient air. Black trace, scan rate 25 mv/sec; red trace, 50 mv/sec; blue trace, 100 mv/sec; green trace, 200 mv/sec; purple trace, 300 mv/sec, 2 mM, 60 sec, 0.1 M $TBAPF_6$ in acetonitrile.

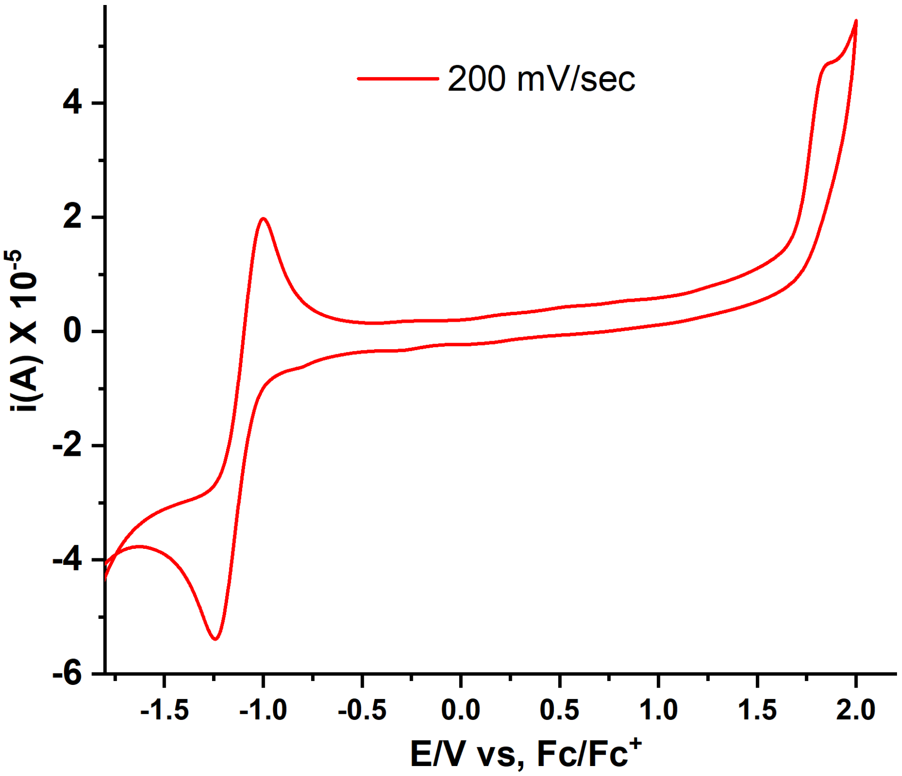

Figure S32. Cyclic voltammograms of benzene **2** on glassy carbon electrodes in ambient air. red trace, scan rate 200 mv/sec, 2 mM, 60 sec, 0.1 M $TBAPF_6$ in acetonitrile.

11. References

1. I. Paul. K. A. Konieczny. R. Chavez. M. A. Garcia-Garibay. Reaction amplification with a gain: Triplet exciton–mediated quantum chain using mixed crystals with a tailor-made triplet sensitizer. Proc. Nat. Acad. Sci. 121, e2401982121 (2024). doi: 10.1073/pnas.2401982121

2 . Bruker (2012). Program name(s). Bruker AXS Inc., Madison, Wisconsin, USA. [Older versions (pre-1997) should refer to Siemens Analytical X-ray Instruments Inc. instead of Bruker AXS.

3. G. M. Sheldrick. A short history of SHELX. Acta Crystallogr., Sect. A: Found. Adv. 64, 112-122 (2008). doi: 10.1107/S0108767307043930

4. G. M. Sheldrick. Crystal structure refinement with SHELXL. Acta Crystallogr., Sect. C: Struct. Chem. 71, 3-8 (2015). doi: 10.1107/S2053229614024218

5. The CCDC #2352506 and #2353011 contains full crystallographic data for this publication. This data can be obtained free of charge from The Cambridge Crystallographic Data Centre via www.ccdc.cam.ac.uk/structures.

6. Rigaku Oxford Diffraction, (2022), CrysAlisPro Software system, version 1.171.42.74a, Rigaku Corporation, Wroclaw, Poland
7. O.V. Dolomanov. L. J. Bourhis. R. J. Gildea. J. A. K. Howard. H. Puschmann. OLEX2: a complete structure solution, refinement and analysis program. J. Appl. Crystallogr. 42, 339−341 (2009). doi: 10.1107/S0021889808042726
8. https://srv.mbi.ucla.edu/faes/ Accessed: April 21, 2024
